# Ceramide(d18:1/18:1)-NDUFA6 interaction inactivates respiratory complex I to attenuate oxidative-stress-driven pathogenesis in liver ischemia/reperfusion injury

**DOI:** 10.1172/jci.insight.187083

**Published:** 2025-04-17

**Authors:** Kai Wang, Leyi Liao, Hanbiao Liang, Pengxiang Huang, Qingping Li, Baoxiong Zhuang, Chen Xie, Xiangyue Mo, Xuesong Deng, Jieyuan Li, Yang Lei, Minghui Zeng, Cungui Mao, Ruijuan Xu, Cuiting Liu, Xianqiu Wu, Jie Zhou, Biao Wang, Yiyi Li, Chuanjiang Li

**Affiliations:** 1 Division of Hepatobiliopancreatic Surgery, Department of General Surgery, Nanfang Hospital, Southern Medical University, Guangzhou, Guangdong, China.; 2 Department of Hepatobiliary Surgery, Shenzhen Third People’s Hospital, Southern University of Science and Technology, Shenzhen, Guangdong, China.; 3 Department of General Surgery, Guangdong Provincial Key Laboratory of Precision Medicine for Gastrointestinal Tumor, Nanfang Hospital, Southern Medical University, Guangzhou, Guangdong, China.; 4 Department of Hepatobiliary Surgery, the First Affiliated Hospital of Shenzhen University, Health Science Center, Shenzhen Second People’s Hospital, Shenzhen, Guangdong, China.; 5 Department of Hepatobiliary Surgery, The First People’s Hospital of Foshan, Foshan, Guangdong, China.; 6 Institute of Scientific Research, Southern Medical University, Guangzhou, China.; 7 Department of Medicine and Cancer Center, The State University of New York at Stony Brook, Stony Brook, New York, USA.; 8 Central Laboratory, and; 9 Department of Radiation Oncology, Nanfang Hospital, Southern Medical University, Guangzhou, Guangdong, China.

**Keywords:** Hepatology, Metabolism, Cell stress, Mitochondria

## Abstract

Oxidative stress driven by malfunctioning respiratory complex I (RC-I) is a crucial pathogenic factor in liver ischemia/reperfusion (I/R) injury. This study investigated the role of alkaline ceramidase 3 (ACER3) and its unsaturated long-chain ceramide (CER) substrates in regulating liver I/R injury through RC-I. Our findings demonstrated that I/R upregulated ACER3 and decreased unsaturated long-chain CER levels in human and mouse livers. Both global and hepatocyte-specific *Acer3* ablation, as well as treatment with CER(d18:1/18:1), led to a significant increase in CER(d18:1/18:1) levels in the liver, which mitigated the I/R-induced hepatocyte damage and inflammation in mice. Mechanistically, ACER3 modulated CER(d18:1/18:1) levels in mitochondria-associated membranes and the endoplasmic reticulum (ER), thereby influencing the transport of CER(d18:1/18:1) from the ER to mitochondria. *Acer3* ablation and CER(d18:1/18:1) treatment elevated CER(d18:1/18:1) in mitochondria, where CER(d18:1/18:1) bound to the RC-I subunit NDUFA6 to inactivate RC-I and reduced reactive oxygen species production in the I/R-injured mouse liver. These findings underscore the role of the CER(d18:1/18:1)-NDUFA6 interaction in suppressing RC-I–mediated oxidative-stress-driven pathogenesis in liver I/R injury.

## Abstract 2

Acer3 ablation and CER(d18:1/18:1) treatment elevate CER(d18:1/18:1) in mitochondria, where CER(d18:1/18:1) binds to the respiratory complex I (RC-I) subunit Ndufa6 to inactivate RC-I and reduce reactive oxygen species production to attenuate liver ischemia/reperfusion injury

## Introduction

Ischemia/reperfusion (I/R) injury is a pathophysiological process caused by cessation and subsequent restoration of blood supply. The liver is particularly susceptible to I/R injury in clinical settings, which frequently occurs in liver resection, liver transplantation, and portal vein embolization, leading to severe consequences, including extensive massive liver damage, liver failure, and even multiple organ dysfunction (1, 2). Despite extensive research, the complex and molecularly obscure mechanisms underlying liver I/R injury have impeded the development of effective therapeutic approaches.

Oxidative stress, induced by a burst of reactive oxygen species (ROS) generation, is the pivotal pathogenic mechanism of liver I/R injury (3, 4). ROS initiates oxidative stress to trigger a cascade of intracellular pathogenesis in I/R injury, including mitochondrial dysfunction, DNA damage, and endoplasmic reticulum (ER) stress, leading to hepatocyte damage and inflammatory response (3, 4). Mitochondria (MT) are the primary source of ROS in hepatocytes, with respiratory complex I (RC-I) being the major producer of deleterious ROS that ignites liver I/R injury (5–8). RC-I is a multisubunit enzyme complex embedded in the mitochondrial inner membrane (9). Under hepatic I/R stress, the production of ROS by RC-I has recently been linked to reverse electron transport on RC-I (5, 10). Inhibitors targeting RC-I, such as rotenone, amobarbital, MitoSNO, and metformin, have shown therapeutic potential in alleviating oxidative-stress-driven pathogenesis in I/R injury (4, 7, 11). Notably, modulating the structure of RC-I to inhibit its catalytic activity of reverse electron transport also reduces ROS generation and alleviates I/R injury (8). However, much remains unclear about the endogenous molecules that interact with RC-I to regulate ROS production, particularly regarding which RC-I subunit is therapeutically targetable for liver I/R injury.

Ceramides (CERs) are bioactive lipids that regulate several pathological processes related to liver I/R injury, including hepatocyte death, hepatic inflammation, and oxidative stress (12). CERs consist of a sphingosine (SPH) backbone linked to a fatty acid with varying acyl-chain lengths and degrees of saturation (12). CERs are produced via the de novo, catabolic, and salvage pathways (12). Upon generation, CERs are hydrolyzed by ceramidases to produce SPH and fatty acids, with SPH further phosphorylated to generate sphingosine-1-phosphate (S1P) (13). Studies have reported that the production of CERs is upregulated in response to experimental hepatic I/R, leading to their accumulation in the liver in murine models (14–18). CERs play important roles in modulating oxidative stress by disrupting mitochondrial membranes, promoting the release of cytochrome *c* and the production of ROS (14, 15). Additionally, CERs interact with various signaling pathways related to mitochondrial oxidative stress, including those regulating antioxidant enzymes and inflammatory mediators, thereby exacerbating oxidative damage and I/R injury (19, 20). Due to these detrimental effects, CERs were previously considered a harmful lipid category in the context of I/R injury (21, 22). However, emerging evidence indicates that the bioactive functions of CER can vary depending on the acyl chains and their metabolizing enzymes, with certain very-long-chain CERs being essential for maintaining physiological homeostasis in the liver (12, 23). The roles of different CERs in mitochondrial pathophysiology are still being intensively investigated. For instance, Hammerschmidt et al. found that CER(d18:1/16:0) derived specifically from ceramide synthase 6, but not ceramide synthase 5, binds to mitochondrial fission factor to induce mitochondrial fragmentation (23), highlighting the distinct function of specific CERs in regulating mitochondrial pathophysiology.

Alkaline ceramidase 3 (ACER3) specifically catalyzes the hydrolysis of unsaturated long-chain CERs (ULCCs), particularly CER(d18:1/18:1) (24, 25). Our previous study reported that targeting ACER3 increases CER(d18:1/18:1) and suppresses oxidative stress in fatty liver (25), but the molecular mechanisms remain unclear. More recently, our study showed that ACER3 and its ULCC substrates are dysregulated in the liver with I/R injury (16, 17). To this end, we thoroughly investigated the role and molecular mechanisms of ACER3 and its ULCC substrates in regulating oxidative stress in liver I/R injury. Our findings reveal that ACER3 regulates the interaction of CER(d18:1/18:1) with NADH-ubiquinone oxidoreductase subunit A6 (NDUFA6) in RC-I to control the I/R-induced ROS production and oxidative stress, thereby impacting the outcomes of liver I/R injury.

## Results

### I/R upregulates ACER3-catalyzed ULCC hydrolysis in human and mouse liver.

**Figure 1:**
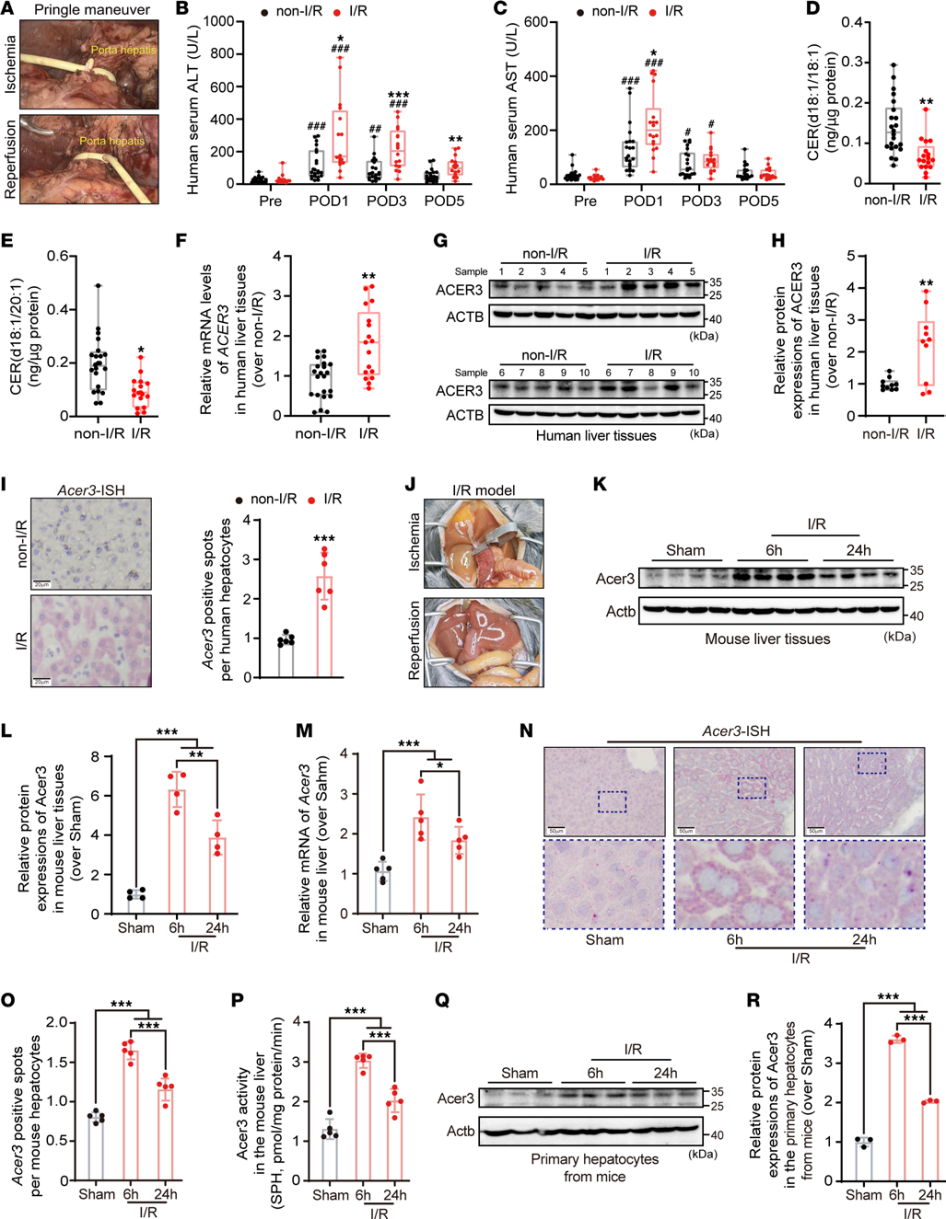
I/R activates ACER3-catalyzed ULCC hydrolysis in human and mouse liver. (**A**) PM performed using a Foley tube to control bleeding during hepatectomy. (**B**–**I**) Evaluation of liver function and ACER3-related metabolism in patients who underwent hepatectomy (*n* = 22 in the non-I/R group, and 17 in the I/R group). Serum ALT (**B**) and AST (**C**) levels in patients measured prior to operation and on postoperative days. Hepatic levels of CER(d18:1/18:1) (**D**) and CER(d18:1/20:1) (**E**) in human liver tissues. Relative mRNA levels (**F**) and protein levels (**G**) of ACER3, and quantification of ACER3 protein levels (**H**) in human liver tissues. (**I**) Representative images of ISH for *ACER3* and quantification of *ACER3*-positive spots in human liver sections. (**J**) Representative images of mouse liver subjected to 1 hour of ischemia followed by reperfusion. (**K**–**R**) Evaluation of Acer3-related expression in mice subjected to IRI (*n* = 5). Relative protein levels (**K**) of Acer3, quantification of Acer3 protein levels (**L**), and *Acer3* mRNA levels (**M**) in mouse liver tissues. Representative images of ISH for *Acer3* (**N**) and quantification of *Acer3*-positive spots (**O**) in mouse liver sections. (**P**) Acer3 activity in mouse liver measured by the production of SPH from hydrolysis of CER(d18:1/18:1). Relative protein levels (**Q**) and quantification of Acer3 (**R**) in hepatocytes isolated from the mouse liver. Scale bars: 20 μm (**I**) and 50 μm (**N**). Data in **B**–**F**, **H**, **I**, **L**, **M**, **O**, **P**, and **R** are expressed as mean ± SD. Statistical significance was determined using 2-tailed Student’s *t* test (**B**–**F**, **H**, and **I**) or 1-way ANOVA followed by Tukey’s test for multiple comparisons (**L**–**M**, **O**, **P**, and **R**). *P < 0.05; **P < 0.01; ***P < 0.001. ^#^*P* < 0.05, ^##^*P* < 0.01, ^###^*P* < 0.001 for comparisons between the postoperative and preoperative levels of ALT and AST (**B** and **C**).

To explore the link between liver I/R injury and CER dysregulation, we measured CERs and their metabolites in normal liver tissues obtained from patients who underwent partial hepatectomy with or without the Pringle maneuver (PM). The PM was performed intermittently using a Foley tube for bleeding control (26), which resulted in liver I/R injury as an adverse effect (Figure 1A). We included 22 patients without PM (non-I/R group) and 17 patients with PM-induced liver injury (I/R group) (Supplemental Table 1; supplemental material available online with this article; https://doi.org/10.1172/jci.insight.187083DS1). Serum transaminase levels, including alanine transaminase (ALT) and aspartate transaminase (AST), were significantly higher in the I/R group than in the non-I/R group after operation (Figure 1, B and C), indicating hepatocyte damage after the PM. CER measurement revealed significant decreases in hepatic ULCCs in the I/R group, including CER(d18:1/18:1) and CER(d18:1/20:1) (Figure 1, D and E). However, no significant differences were observed in other detected CER species, SPH, or S1P levels in liver tissues (Supplemental Figure 1, A–L). These findings suggest that intermittent I/R procedures result in decreased ULCC levels in the human liver. ACER3 specifically catalyzes the degradation of ULCCs (24, 25). In line with the decreased hepatic ULCCs by I/R, we found that the mRNA and protein levels of *ACER3* were significantly increased in liver tissues in the I/R group (Figure 1, F–H). In situ hybridization (ISH) revealed that *ACER3* mRNA levels were significantly increased in hepatocytes in the I/R group (Figure 1I). To further investigate the role of ACER3 and ULCCs in liver I/R injury, we established a mouse model of warm liver I/R injury, involving 1 hour of ischemia followed by 6 and 24 hours of reperfusion (Figure 1J). I/R significantly increased both mRNA and protein levels of Acer3 in mouse liver tissues (Figure 1, K–M). Notably, Acer3/*Acer3* expression in mouse livers was significantly upregulated at 6 hours of reperfusion, followed by a downward trend at 24 hours (Figure 1, K–M). Similarly, enzymatic activities of Acer3 in mouse liver were also upregulated after I/R (Figure 1P). ISH also revealed that *Acer3* mRNA levels were significantly increased in hepatocytes in the mouse liver following I/R injury (Figure 1, N and O). Consistently, measurement of Acer3 protein levels in hepatocytes isolated from the mouse liver demonstrated that I/R significantly increased Acer3 protein in mouse hepatocytes (Figure 1, Q and R). These findings demonstrated that I/R induces ULCC reduction and upregulates *ACER3* in human and mouse livers, suggesting a role for overactive ACER3-catalyzed ULCC hydrolysis in liver I/R injury.

### Global and hepatocyte-specific Acer3 ablation increases CER(d18:1/18:1) to attenuate liver I/R injury.

**Figure 2:**
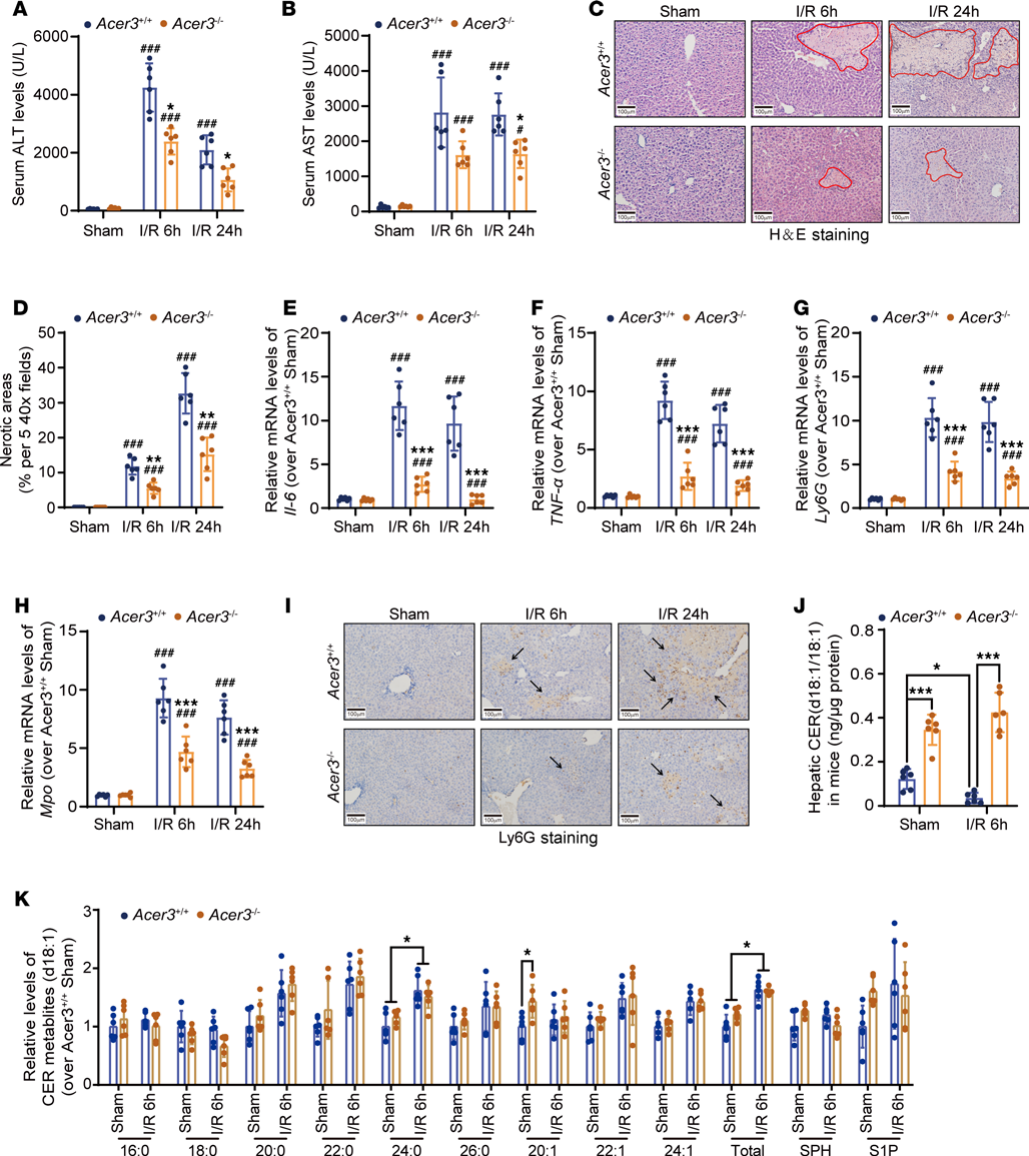
*Acer3* ablation attenuates liver I/R injury and increases hepatic CER(d18:1/18:1) in male mice. (**A** and **B**) Serum ALT (**A**) and AST (**B**) levels in *Acer3^+/+^* and *Acer3^–/–^* male mice after sham operation and at 6 hours and 24 hours after I/R (*n* = 6). (**C** and **D**) Representative H&E staining of liver sections, with circled areas indicating necrotic foci (**C**), and quantification of necrotic area (**D**) in *Acer3^+/+^* and *Acer3^–/–^* male mice after sham operation and at 6 hours and 24 hours after I/R (*n* = 6). (**E**–**I**) Relative mRNA levels of proinflammatory cytokines *Il-6* (**E**) and *Tnf-α* (**F**) and neutrophil markers *Ly6g* (**G**) and *Mpo* (**H**) in liver tissues from *Acer3^+/+^* and *Acer3^–/–^* male mice after sham operation and at 6 hours and 24 hours after I/R. Representative Ly6G staining of liver sections, with black arrows indicating inflammatory infiltration after sham operation and at 6 hours and 24 hours after I/R (**I**) (*n* = 6). (**J** and **K**) CER(d18:1/18:1) levels (**J**) and relative levels of CERs and CER metabolites (**K**) in the liver tissues from *Acer3^+/+^* and *Acer3^–/–^* male mice after sham operation and 6 hours after I/R (*n* = 6). Scale bars: 100 μm. Data in **A**, **B**, **D**–**H**, **J**, and **K** are expressed as mean ± SD. Statistical significance was determined using 1-way ANOVA followed by Tukey’s test for multiple comparisons. *P < 0.05; **P < 0.01; ***P < 0.001. ^###^*P* < 0.001 for comparisons between sham and I/R (**A**, **B**, and **D**–**H**).

To elucidate the role of ACER3 and ULCCs in liver I/R injury, we investigated the impacts of *Acer3* knockout (KO) on experimental liver I/R injury in mice. In the mouse model of hepatic I/R injury, the 6-hour time point was chosen to examine early molecular mechanisms characterized by pronounced inflammation and transaminase leakage, while the 24-hour time point reflects a later phase with ongoing damage and the initiation of recovery processes (27–29). Accordingly, our data showed that serum transaminase levels and inflammatory responses markedly increased at 6 hours of reperfusion and declined by 24 hours, whereas liver necrosis progressively expanded at 24 hours (Figure 2, A–I). Interestingly, *Acer3^–/–^* male mice exhibited significantly lower serum transaminase levels and reduced liver necrosis compared with their wild-type littermates (*Acer3^+/+^* mice) after experimental liver I/R injury (Figure 2, A–D). Additionally, *Acer3^–/–^* male mice showed decreased inflammatory responses, evidenced by lower cytokine expression and reduced neutrophil infiltration (Figure 2, E–I). Similarly, *Acer3^–/–^* female mice exhibited alleviative I/R-induced liver injury (Supplemental Figure 2, A–I). These findings suggest that *Acer3* ablation attenuated liver I/R injury in mice regardless of sex. Since we observed upregulation of ACER3/*Acer3* in hepatocytes following I/R injury, we investigated the impact of hepatocyte-specific *Acer3* ablation in liver I/R injury. Hepatocyte-specific *Acer3*-KO (*Acer3*^ΔHep^) mice were established with the Cre-*loxP* system by deleting exons 3 and 4 of the *Acer3* gene (Supplemental Figure 3, A and B). In these mice, the I/R-induced upregulation of *Acer3* expression was markedly reduced (Supplemental Figure 3C). Similar to observations in global *Acer3*-KO mice, hepatocyte-specific *Acer3* deficiency significantly protected against hepatic I/R injury, as evidenced by reduced liver dysfunction, necrosis, and inflammation (Supplemental Figure 3, D–L). These findings emphasize the pathogenic role of hepatic ACER3 in liver I/R injury and demonstrate the sustained protective effects of targeting ACER3 in the progression of liver I/R injury.

**Figure 3:**
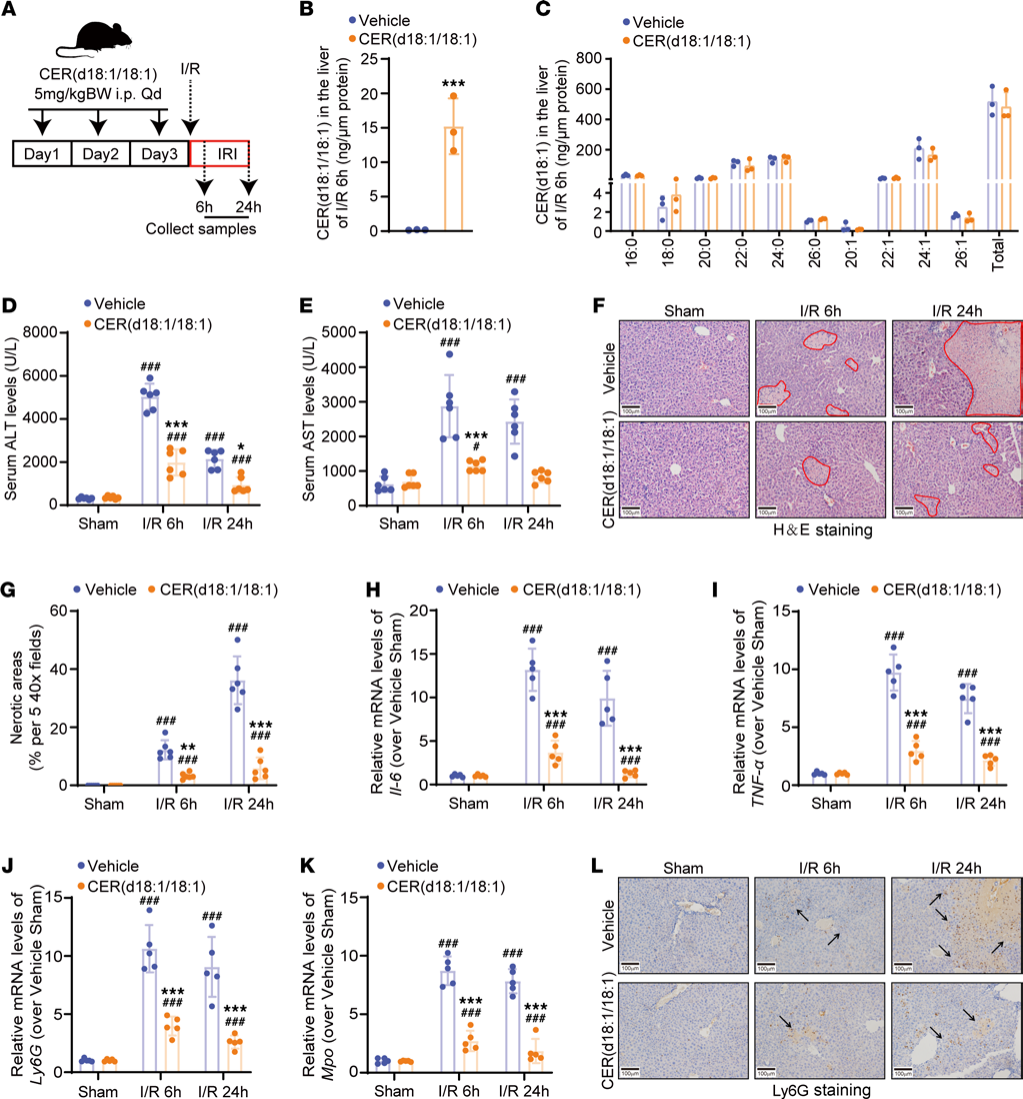
CER(d18:1/18:1) treatment attenuates liver I/R injury. (**A**) Schematic of the experimental design for CER(d18:1/18:1) treatment in male mice subjected to I/R injury. C57BL/6J wild-type mice were intraperitoneally treated with CER(d18:1/18:1) (5 mg/kg BW, once per day) before and after I/R injury. (**B** and **C**) CER(d18:1/18:1) levels (**B**) and other CER levels (**C**) in the vehicle- and CER(d18:1/18:1)-treated mice after sham operation and at 6 hours after I/R (*n* = 3). (**D** and **E**) Serum ALT (**D**) and AST (**E**) levels in the vehicle- and CER(d18:1/18:1)-treated mice after sham operation and at 6 hours and 24 hours after I/R (*n* = 6). (**F** and **G**) Representative H&E staining of liver sections, with circled areas indicating necrotic foci (**F**), and quantification of necrotic area (**G**) in the vehicle- and CER(d18:1/18:1)-treated mice after sham operation and at 6 hours and 24 hours after I/R (*n* = 6). (**H**–**L**) Relative mRNA levels of proinflammatory cytokines *Il-6* (**H**) and *Tnf-α* (**I**) and neutrophil markers *Ly6g* (**J**) and *Mpo* (**K**) in liver tissues from vehicle- and CER(d18:1/18:1)-treated mice after sham operation and at 6 hours and 24 hours after I/R. (**L**) Representative Ly6G staining of liver sections, with black arrows indicating inflammatory infiltration after sham operation and at 6 hours and 24 hours after I/R (*n* = 5). Scale bars: 100 μm. Data in **B**–**E** and **G**–**K** are expressed as mean ± SD. Statistical significance was determined using 2-tailed Student’s *t* test (**B** and **C**) or 1-way ANOVA followed by Tukey’s test for multiple comparisons (**D**–**E** and **G**–**K**). *P < 0.05; **P < 0.01; ***P < 0.001. ^#^*P* < 0.05, ^###^*P* < 0.001 for comparisons between sham and I/R (**D**, **E**, and **G**–**K**).

CER measurements showed that I/R significantly decreased hepatic CER(d18:1/18:1) while increasing CER(d18:1/24:0) and total CER levels in mice (Figure 2, J and K, and Supplemental Figure 4, A–L). In addition to *Acer3*, I/R upregulated mRNA and protein levels of CER biosynthetic enzymes, including ceramide synthases (e.g., Cers2) and sphingomyelinase (e.g., Smpd3) (Supplemental Figure 4, M–R), indicating that I/R promoted CER production in the mouse liver. ACER3 is known to specifically catalyze the hydrolysis of ULCCs, particularly CER(d18:1/18:1) (24, 30). Accordingly, CER measurements revealed that *Acer3* ablation specifically increased CER(d18:1/18:1) and prevented the I/R-induced decrease in CER(d18:1/18:1) (Figure 2, J and K, and Supplemental Figure 4, A–L). Given that *Acer3* ablation prevented the I/R-induced decrease in CER(d18:1/18:1) and attenuated liver I/R injury, we examined whether CER(d18:1/18:1) treatment attenuates liver I/R injury in C57BL/6J wild-type mice (Figure 3A). CER(d18:1/18:1) treatment significantly increased the levels of CER(d18:1/18:1) in the liver after I/R (Figure 3, B and C). Similar to *Acer3* ablation, CER(d18:1/18:1) treatment alleviated liver I/R injury, demonstrated by lower serum transaminases, reduced necrotic areas, and suppressed inflammation, as shown by lower cytokine expression and reduced neutrophil infiltration at both 6- and 24-hour reperfusion time points (Figure 3, D–L). These results demonstrated that *Acer3* ablation increases CER(d18:1/18:1) to attenuate liver I/R injury, suggesting that Acer3-catalyzed CER(d18:1/18:1) hydrolysis plays a pathogenic role in liver I/R injury.

### CER(d18:1/18:1) interacts with RC-I subunit Ndufa6 in MT in the liver with I/R injury.

**Figure 4:**
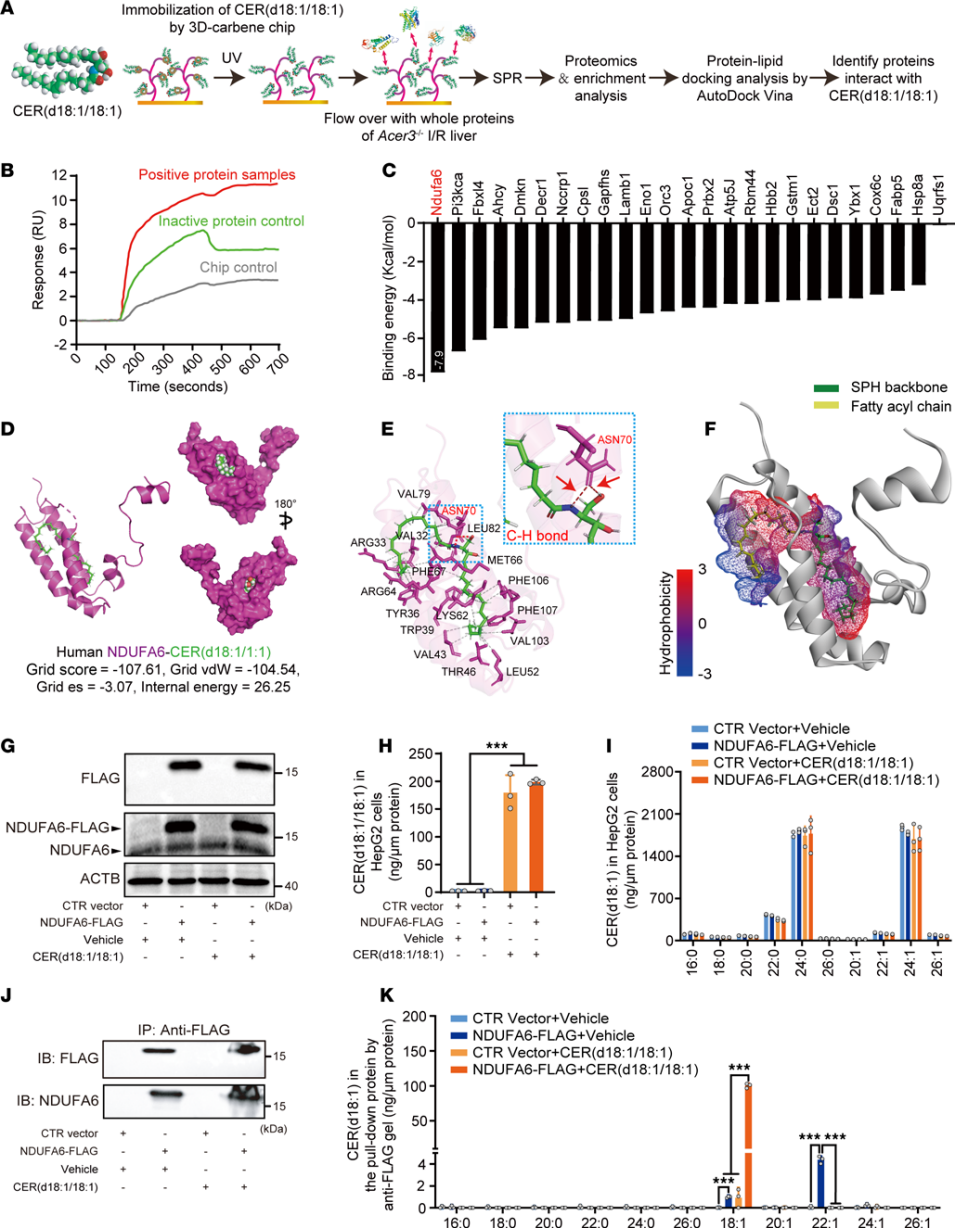
CER(d18:1/18:1) interacts with RC-I subunit NDUFA6. (**A**) Schematic of the experimental workflow for identifying CER(d18:1/18:1)-binding proteins using 3D carbene chip SPR, proteomics, and protein-lipid docking analysis with AutoDock Vina (see Supplemental Methods). (**B**) SPR analysis showing the binding response of CER(d18:1/18:1)-binding proteins in positive protein samples, inactive protein control, and chip control. (**C**) Predicted binding energy of CER(d18:1/18:1) with captured proteins, highlighting the highest binding affinity for Ndufa6. (**D**–**F**) Model of the CER(d18:1/18:1)–human NDUFA6 complex (**D**), interaction details (**E**), and hydrophobicity analysis (**F**). vdW, van der Waals; es, electrostatic. (**G**) Immunoblot analysis showing the overexpression of FLAG-tagged NDUFA6 in HepG2 cells treated with CER(d18:1/18:1). (**H** and **I**) Levels of CER(d18:1/18:1) (**H**) and other CER species in HepG2 cells overexpressing NDUFA6-FLAG (**I**). (**J** and **K**) Immunoprecipitation of FLAG-tagged NDUFA6 in HepG2 cells (**J**) and levels of CER(d18:1/18:1) in immunoprecipitated NDUFA6 protein from HepG2 cells (**K**). Images in **G**–**K** represent the results of 3 independent experiments. Data in **H**, **I**, and **K** are expressed as mean ± SD. Statistical significance was determined using 1-way ANOVA followed by Tukey’s test for multiple comparisons. ***P < 0.001.

To dissect the molecular mechanism by which CER(d18:1/18:1) alleviates liver I/R injury, we performed surface plasmon resonance (SPR) with 3D carbene chips, proteomics, and in silico protein-lipid docking to identify proteins interacting with CER(d18:1/18:1) in the liver with I/R injury (Figure 4A) (31, 32). CER(d18:1/18:1) was immobilized on the 3D carbene chip to capture binding proteins extracted from the livers of *Acer3^–/–^* mice with I/R injury. SPR detected significant signals reflecting the interactions between captured proteins and the immobilized CER(d18:1/18:1) (Figure 4B). The captured proteins were then identified by proteomics (Supplemental Table 3), and docking analysis revealed that Ndufa6 had the highest predicted binding affinity for CER(d18:1/18:1) (Figure 4C). NDUFA6 features a hydrophobic crevice between its helices that interacts with acyl chains (9, 33). To further validate the interaction of CER(d18:1/18:1)-NDUFA6, we modeled the CER(d18:1/18:1)-NDUFA6 complex virtually. The model revealed similar structures and strong binding affinities for the CER(d18:1/18:1)-NDUFA6 interaction in both mice (Supplemental Figure 5, A and B) and humans (Figure 4, D and E), showing that the fatty acyl chain or the SPH backbone of CER(d18:1/18:1) inserted into the hydrophobic crevice of NDUFA6 (Figure 4F and Supplemental Figure 5C), interacting with NDUFA6 through carbon-hydrogen bonds, van der Waals, and alkyl interactions (Figure 4E and Supplemental Figure 5, B and D). Next, we precipitated the FLAG-tagged NDUFA6 overexpressed in HepG2 cells treated with CER(d18:1/18:1) in a detergent-free manner and measured CER levels in the precipitated NDUFA6 protein. CER(d18:1/18:1) treatment increased CER(d18:1/18:1) levels in HepG2 cells without affecting the protein levels of NDUFA6 (Figure 4, G–I). CER(d18:1/18:1) and CER(d18:1/22:1) were detected in precipitated NDUFA6, with CER(d18:1/18:1) significantly increasing upon treatment with CER(d18:1/18:1) (Figure 4, J and K). By in silico analysis of affinities between NDUFA6 and other CERs, we found that unsaturated CERs had higher predicted affinities for NDUFA6 than saturated CERs, with ULCCs in particular exhibiting the highest affinities (Supplemental Figure 5E). Functional protein association network analysis using the STRING online tool (https://cn.string-db.org/) revealed that proteins Atp5j, Cox6c, and Uqcrfs1, captured alongside Ndufa6 on the CER(d18:1/18:1)-immobilized chip, are functionally associated and involved in oxidative phosphorylation and electron transport chain pathways (Supplemental Figure 5, F and G), suggesting the CER(d18:1/18:1)-NDUFA6 interaction potentially regulates these pathways to affect liver I/R injury.

### Acer3 ablation increases mitochondrial CER(d18:1/18:1) to inhibit RC-I activity in I/R-injured liver.

**Figure 5:**
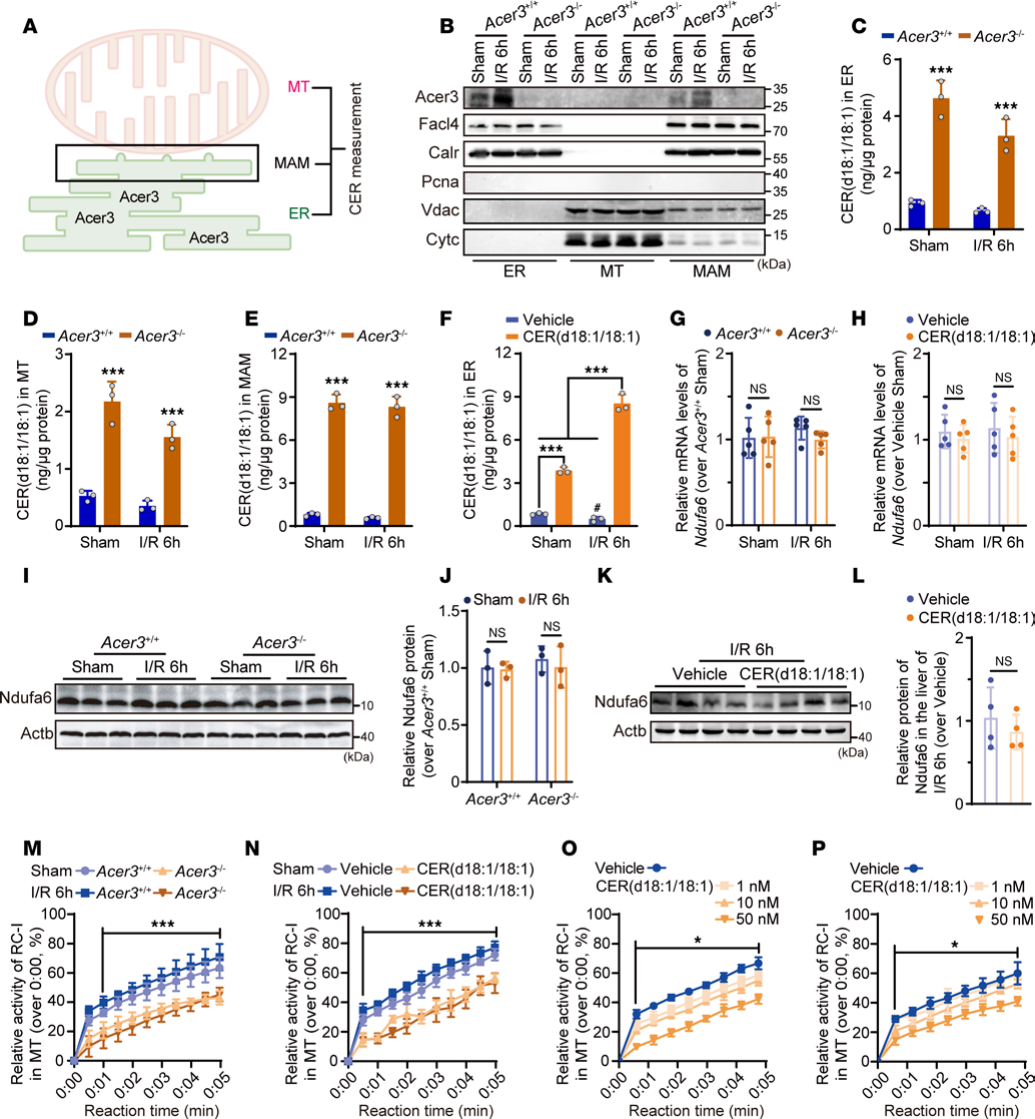
*Acer3* ablation and CER(d18:1/18:1) treatment inhibit RC-I activity in the liver with I/R injury. (**A**) Schematic of the experimental design showing the location of Acer3 and CER measurement in ER, MAM, and MT fractions in liver tissues. (**B**) Immunoblot analysis confirming the isolation and purification of ER, MAM, and MT fractions using specific MAM marker FACL4, ER marker CALR, nuclear marker PCNA, and MT markers VDAC and CYC. The protein levels of Acer3 were measured in these fractions. (**C**–**E**) CER(d18:1/18:1) levels in the ER (**C**), MT (**D**), and MAMs (**E**) from liver tissues of *Acer3^+/+^* and *Acer3^–/–^* mice after sham operation and at 6 hours after I/R (*n* = 3). (**F**) CER(d18:1/18:1) levels in MT from mice treated with vehicle or CER(d18:1/18:1) after sham operation and at 6 hours after I/R (*n* = 3). (**G**–**L**) Ndufa6 expression in liver tissues from *Acer3^+/+^* and *Acer3^–/–^* mice and vehicle- and CER(d18:1/18:1)-treated mice after sham operation and at 6 hours after I/R (*n* = 5). (**G** and **H**) Relative mRNA levels of *Ndufa6* in liver tissues. (**I** and **K**) Immunoblot analysis of Ndufa6 protein levels and (**J** and **L**) quantification of Ndufa6 protein levels in liver tissues. (**M**–**P**) RC-I activity in liver tissues from *Acer3^+/+^* and *Acer3^–/–^* mice (**M**) and vehicle- and CER(d18:1/18:1)-treated mice (**N**) after sham operation and at 6 hours after I/R (*n* = 4). RC-I activity in mouse (**O**) and human MT (**P**) treated with different concentrations of CER(d18:1/18:1) (*n* = 3). Images in **B** represent the results of 3 independent experiments. Data in **C**–**H**, **J**, **L**, and **M**–**P** are expressed as mean ± SD. Statistical significance was determined using 2-tailed Student’s *t* test (**L**) or 1-way ANOVA followed by Tukey’s test for multiple comparisons (**C**–**H**, **J** and **M**–**P**). *P < 0.05; ***P < 0.001.

NDUFA6 is a critical subunit of RC-I in MT (9, 33). ACER3 is a Golgi and ER protein (24). MT-associated ER membranes (MAMs) are known to transfer lipids between the ER and MT (23, 34). To confirm that ACER3 is capable of regulating the CER(d18:1/18:1)-NDUFA6 interaction in MT, we first determined whether ACER3 likely regulates the CER(d18:1/18:1) levels in MT via lipid transfer from the ER through MAMs (Figure 5A). Isolation and purification of these fractions from mouse liver were confirmed by immunoblotting of corresponding markers (Figure 5B). Notably, we observed that Acer3 protein was expressed in the ER and MAM fractions (Figure 5B). CER measurement demonstrated that I/R significantly increased the levels of total CER, SPH, and/or S1P in the ER and MAMs (Figure 5, C–E), while decreasing total CER levels in the MT of the mouse liver (Supplemental Figure 6). I/R also decreased the levels of CER(d18:1/18:1) in the liver ER, MAMs, and MT in *Acer3^+/+^* mice (Figure 5, C–E). In contrast, *Acer3* ablation specifically increased CER(d18:1/18:1) levels in the ER and MT under basal conditions and I/R conditions (Supplemental Figure 6, A–D). Interestingly, *Acer3* ablation increased most CER species and SPH in MAMs under normal and I/R conditions (Supplemental Figure 6, E and F). Intraperitoneal administration of CER(d18:1/18:1) also increased CER(d18:1/18:1) levels in MT without significantly affecting other CER species, SPH, or S1P (Figure 5F and Supplemental Figure 6, G and H). These findings suggested that ACER3 catalyzes the degradation of CER(d18:1/18:1) within both the ER and MAMs, thereby regulating its levels in these compartments. This modulation of CER(d18:1/18:1) content in the ER and MAMs may, in turn, influence CER(d18:1/18:1) transport from the ER to MT via the MAMs.

To further explore the consequence of this interaction, we assessed whether *Acer3* ablation or CER(d18:1/18:1) administration affects *Ndufa6* expression in I/R-injured liver. We found no changes in mRNA and protein levels of Ndufa6 following Acer3 ablation or CER(d18:1/18:1) administration (Figure 5, G–L). Given that Ndufa6 is a subunit of RC-I that is essential for maintaining the activity of RC-I (9, 33), we examined the impact of *Acer3* ablation and CER(d18:1/18:1) administration on RC-I activity. Both *Acer3* ablation and CER(d18:1/18:1) administration significantly suppressed RC-I activity in the normal and I/R-injured livers (Figure 5, M and N). Furthermore, CER(d18:1/18:1) treatment directly suppressed RC-I activity in MT isolated from mouse liver and HepG2 cells (Figure 5, O and P). These findings demonstrated that ACER3 regulates RC-I activity by controlling CER(d18:1/18:1) levels, which interact with NDUFA6 in RC-I.

### Acer3 ablation increases CER(d18:1/18:1) to reduce oxidative stress by promoting CER(d18:1/18:1)-Ndufa6 interaction in I/R-injured liver I/R.

**Figure 6:**
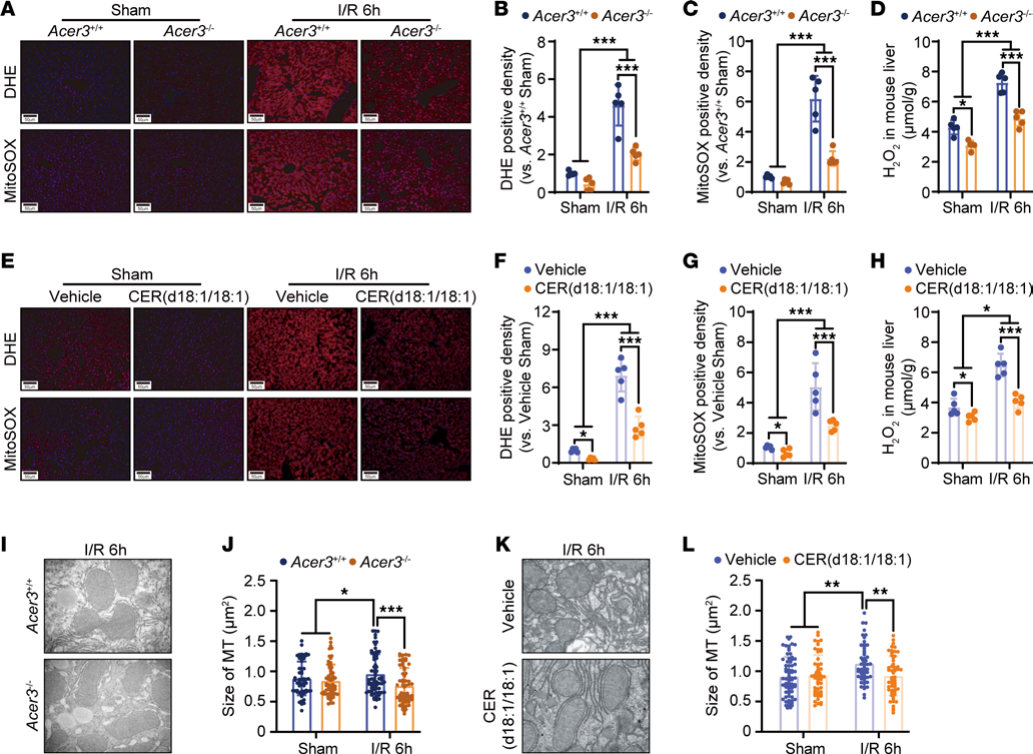
*Acer3* ablation and CER(d18:1/18:1) treatment reduce ROS production and alleviate MT damage in liver I/R injury. (**A**) Representative images of DHE and MitoSOX staining in liver sections from *Acer3^+/+^* and *Acer3^–/–^* mice after sham operation and 6 hours after I/R (*n* = 5). (**B** and **C**) Quantification of DHE staining density (**B**) and MitoSOX staining density (**C**) in liver sections from *Acer3^+/+^* and *Acer3^–/–^* mice after sham operation and at 6 hours after I/R (*n* = 5). (**D**) Levels of H_2_O_2_ in liver tissues from *Acer3^+/+^* and *Acer3^–/–^* mice after sham operation and at 6 hours after I/R (*n* = 5). (**E**) Representative images of DHE and MitoSOX staining in liver sections from the vehicle- and CER(d18:1/18:1)-treated mice after sham operation and at 6 hours after I/R (*n* = 5). Scale bars: 50 μm (**A** and **E**). (**F** and **G**) Quantification of DHE staining density (**F**) and MitoSOX staining density (**G**) in liver sections from the vehicle- and CER(d18:1/18:1)-treated mice after sham operation and at 6 hours after I/R (*n* = 5). (**H**) Levels of H_2_O_2_ in liver tissues from the vehicle- and CER(d18:1/18:1)-treated mice after sham operation and at 6 hours after I/R (*n* = 5). (**I**–**L**) Representative TEM images and quantification of mitochondrial size in liver tissues from *Acer3^+/+^* and *Acer3^–/–^* mice (**I** and **J**) and vehicle- and CER(d18:1/18:1)-treated mice (**K** and **L**) after sham operation and at 6 hours after I/R (*n* = 5). Original magnification, ×15,000 (**I** and **K**). Data in **B**–**D**, **F**–**H**, **J**, and **L** are expressed as mean ± SD. Statistical significance was determined using 1-way ANOVA followed by Tukey’s test for multiple comparisons. *P < 0.05; **P < 0.01; ***P < 0.001.

RC-I critically regulates oxidative stress through ROS production (5). Since *Acer3* ablation increased CER(d18:1/18:1) and inhibited RC-I activity, we investigated its impact on oxidative stress in I/R-injured liver at the 6-hour perfusion time point. Utilizing dihydroethidium (DHE), MitoSOX, and hydrogen peroxide to measure ROS, we found that *Acer3* ablation significantly reduced I/R-induced ROS production in the mouse liver (Figure 6, A–D). Similarly, ROS production was decreased in the liver of CER(d18:1/18:1)-treated mice following I/R injury (Figure 6, E–H). Moreover, transmission electron microscopy (TEM) examination demonstrated that *Acer3* ablation and CER(d18:1/18:1) treatment attenuated the I/R-induced mitochondrial swelling and MT damage in mouse liver (Figure 6, I–L). These findings confirmed that *Acer3* ablation increases CER(d18:1/18:1) to prevent ROS production and MT damage in I/R-injured liver.

**Figure 7:**
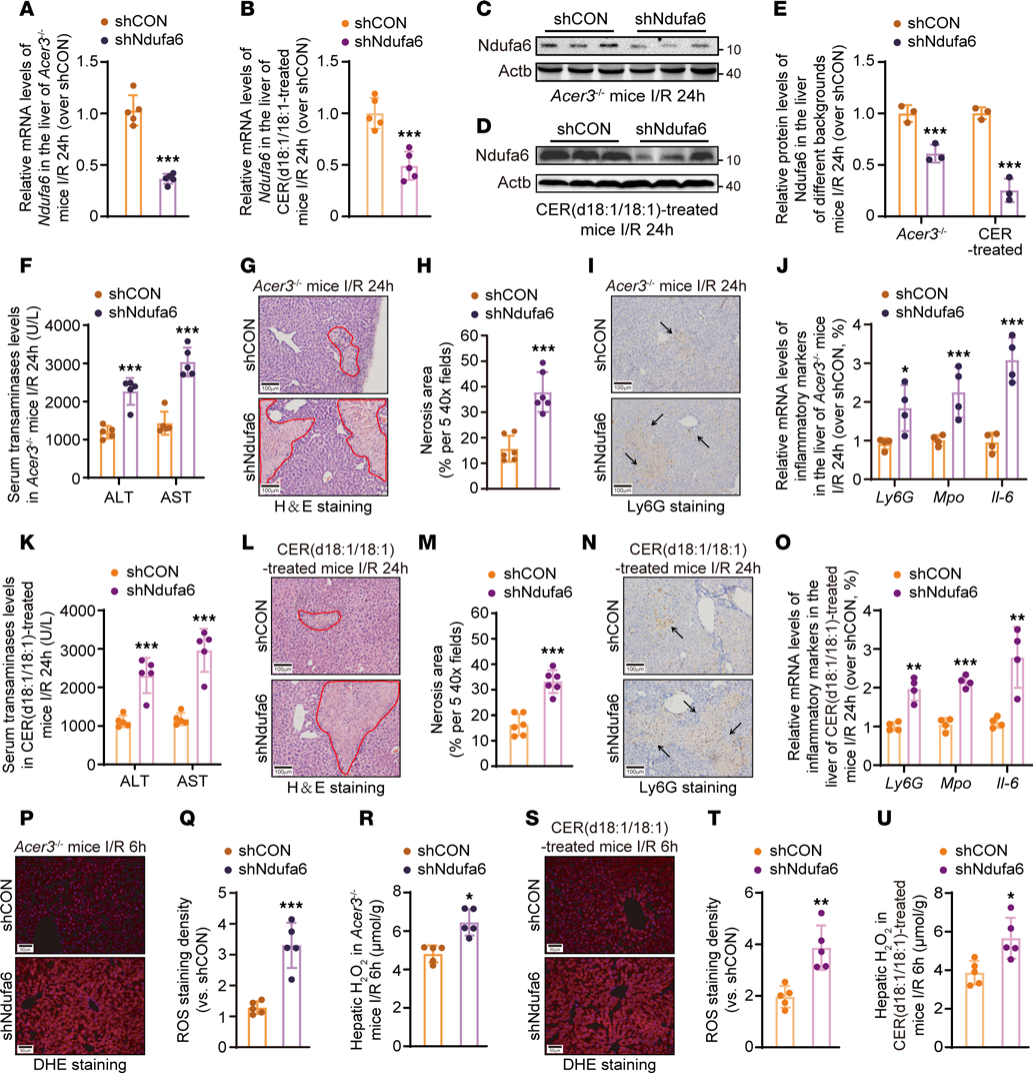
*Ndufa6* knockdown impairs the protective functions of *Acer3* ablation and CER(d18:1/18:1) treatment against liver I/R injury. (**A**–**E**) Ndufa6 expression in liver tissues from shCON- and shNdufa6-AAV–transfected *Acer3^–/–^* mice and CER(d18:1/18:1)-treated mice after sham operation and at 24 hours after I/R (*n* = 5). (**A** and **B**) Relative mRNA levels and immunoblot analysis of *Ndufa6*. (**C** and **D**) Immunoblot analysis of Ndufa6 protein levels and (**E**) quantification of Ndufa6 protein levels. (**F**–**J**) Pathological evaluation in shCON- and shNdufa6-AAV–transfected *Acer3^–/–^* mice after sham operation and at 24 hours after I/R (*n* = 5). (**F**) The levels of serum transaminases. Representative H&E staining of liver sections, with circled areas indicating necrotic foci (**G**), and quantification of necrotic area (**H**). Representative Ly6G staining, with black arrows indicating inflammatory infiltration (**I**) and relative mRNA levels of inflammatory markers (*Ly6g*, *Mpo*, and *Il-6*) (**J**). (**K**–**O**) Pathological evaluation in shCON- and shNdufa6-AAV–transfected C57BL/6J wild-type mice with CER(d18:1/18:1) treatment after sham operation and at 24 hours after I/R (*n* = 5). (**K**) The levels of serum transaminases. Representative H&E staining of liver sections, with circled areas indicating necrotic foci (**L**), and quantification of necrotic area (**M**). Representative Ly6G staining with black arrows indicating inflammatory infiltration (**N**), and relative mRNA levels of inflammatory markers (*Ly6g*, *Mpo*, and *Il-6*) (**O**). (**P**–**V**) ROS evaluation in liver tissues from shCON- and shNdufa6-AAV-transfected *Acer3^–/–^* mice and CER(d18:1/18:1)-treated mice after sham operation and at 6 hours after I/R. Representative DHE staining (**P** and **T**), quantification of DHE staining density (**R** and **U**), and the levels of H_2_O_2_ (**S** and **V**). Scale bars: 100 μm (**G**, **I**, **L**, and **N**) and 50 μm (**P** and **S**). Data in **A**, **B**, **E**, **F**, **H**, **J**, **K**, **M**, **O**, **R**, **S**, **U**, and **V** are expressed as mean ± SD. Statistical significance was determined using 2-tailed Student’s *t* test. *P < 0.05, **P < 0.01, ***P < 0.001.

To define the role of NDUFA6 in mediating the protective effects of CER(d18:1/18:1) against I/R-induced liver injury, we knocked down *Ndufa6* (Figure 7, A–E) and tested whether *Ndufa6* knockdown impaired the protective effects of *Acer3* ablation and CER(d18:1/18:1) treatment against liver I/R injury. *Ndufa6* knockdown significantly exaggerated liver I/R injury in *Acer3^–/–^* and CER(d18:1/18:1)-treated mice, indicated by higher serum transaminase levels, larger liver necrotic areas, and aggravated liver inflammatory response with higher inflammatory cytokine expression and increased neutrophil infiltration in *Ndufa6*-knockdown mice compared with the control mice after I/R (Figure 7, F–O). Next, we investigated the impacts of *Ndufa6* knockdown on ROS production. Detection of ROS content revealed that *Ndufa6* knockdown significantly increased ROS production in the liver after hepatic I/R (Figure 7, P–V), confirming that *Ndufa6* knockdown reduced the protective effects of *Acer3* ablation and CER(d18:1/18:1) treatment on suppressing the I/R-induced ROS production. These data indicated that *Ndufa6* is essential for *Acer3* ablation and CER(d18:1/18:1) to protect the liver against liver I/R injury through suppressing oxidative-stress-driven pathogenesis.

## Discussion

This study investigated the role and molecular mechanisms of ACER3 and its ULCC substrates in regulating liver I/R injury. Our findings demonstrated that I/R activates hepatic ACER3-catalyzed ULCC hydrolysis to reduce CER(d18:1/18:1) in human and mouse liver. Increasing CER(d18:1/18:1) through *Acer3* ablation or CER(d18:1/18:1) treatment attenuates liver I/R injury. Mechanistically, CER(d18:1/18:1) interacts with RC-I subunit Ndufa6 to inactivate RC-I and reduce ROS production, thereby attenuating oxidative-stress-driven pathogenesis in liver I/R injury.

Accumulation of CERs, initiated from de novo synthesis and sphingomyelin degradation, has been observed in the mouse liver with experimental I/R injury (14–18). Our study also observed an increase in total CERs in the liver tissues in mice subjected to liver I/R injury (Figure 2K). The generation of CER is regulated by a network of enzymatic pathways, including CER synthases, sphingomyelin degradation, and glycosphingolipid catabolism, as well as the conversion of CER into sphingomyelin or glycosphingolipids (12). These processes collectively regulate the levels of various CER species (35). Our data showed that I/R increases the expression of CER-metabolizing enzymes associated with CER generation, such as CER synthases (e.g., Cers2) and sphingomyelinase (e.g., Smpd3) (Supplemental Figure 4), which catalyzes the biosynthesis of CERs (12) and the degradation of sphingomyelins to produce CER (12), respectively. Therefore, the increase in CER species in the mouse liver with I/R injury may result from upregulated CER generation (Supplemental Figure 4). However, our study revealed no such increase in CERs in I/R-challenged liver tissues in patients (Supplemental Figure 1). This paradoxical alteration in hepatic CERs may result from the differences in I/R procedure between experimental liver I/R injury and clinical operation. Unlike experimental liver I/R injury, which intentionally prolongs the ischemic period to augment the liver injury, clinical operation adopts intermittent I/R procedures to limit consequent injury. Therefore, intermittent I/R procedures might prevent CER accumulation in the liver. Notably, hepatic *ACER3* upregulation and ULCC decrease were observed in both patients and experimental mice after different I/R procedures (Figure 1), indicating *ACER3* expression is sensitively regulated by I/R, and *ACER3* regulation by I/R is conserved. Moreover, *Acer3* ablation was found to abolish the decrease in ULCCs and increase hepatic ULCCs after I/R (Figure 2J), suggesting that I/R upregulates hepatic ACER3 to promote the hydrolysis of ULCCs, leading to the reduction in ULCCs. In the mouse model of hepatic I/R injury, the 6-hour time point was selected to investigate the early molecular mechanisms of I/R injury, as this phase is characterized by pronounced inflammatory responses and transaminase leakage (27, 28). The 24-hour time point represents a later phase of injury, reflecting ongoing damage and the initiation of resolution processes, where inflammation and necrosis begin to recover (29). Accordingly, our findings demonstrated that serum transaminase levels and inflammatory responses were significantly elevated at the 6-hour reperfusion time point but began to decrease by 24 hours (Figure 2, A, B, and E–I). However, liver necrosis expanded progressively, with the larger necrotic areas observed at 24 hours (Figure 2, C and D). Manipulating the ACER3/ULCC metabolic axis by *Acer3* ablation and CER(d18:1/18:1) treatment to increase hepatic CER(d18:1/18:1) significantly alleviated liver I/R injury in both male and female mice (Figures 2 and 3, and Supplemental Figure 2). Notably, *Acer3* upregulation was observed at 6 hours of reperfusion in I/R livers and showed a decreasing trend by 24 hours, although it remained higher than sham levels (Figure 1, K–M). Importantly, *Acer3* deficiency and CER(d18:1/18:1) treatment effectively alleviated liver I/R injury at both 6- and 24-hour time points (Figures 2 and 3). These findings emphasize the pivotal role of ACER3 in early-stage I/R injury through its specific degradation of CER(d18:1/18:1) and demonstrate the sustained protective effects of targeting ACER3 and CER(d18:1/18:1) metabolism throughout the injury progression regardless of sex. However, given the complexity of CER metabolism, it is important to acknowledge that other enzymes, in addition to ACER3, may also play critical roles in regulating CER metabolism during liver I/R injury. For instance, acid ceramidase, SPH kinase, and CER kinase have also been reported to protect liver I/R injury by regulating different CER metabolites (16, 36, 37). These findings collectively suggest that targeting CER metabolism could be an effective therapeutic strategy for liver I/R injury.

*ACER3* expression is known to increase in the liver under pathological conditions such as MAFLD and HCC (17, 25, 38). Our study extends these findings, demonstrating that hepatic ACER3 is also upregulated in response to I/R injury. According to the Human Protein Atlas (https://www.proteinatlas.org/ENSG00000078124-ACER3/single+cell/liver), ACER3 is predominantly expressed in Kupffer cells, vascular endothelial cells, and neutrophils, with relatively low expression in hepatocytes. To investigate the cell-type-specific regulation of *Acer3* under I/R conditions, we performed ISH to localize *Acer3* expression and measured its protein levels in isolated mouse hepatocytes. We found that both mRNA and protein levels of Acer3 were significantly increased in hepatocytes following I/R injury (Figure 1, O–R). These findings indicate that while baseline ACER3 expression is low in hepatocytes, it can be markedly upregulated under disease conditions, suggesting its significant role in the pathogenesis of liver diseases. To further clarify the contribution of hepatocyte-specific *Acer3* in I/R injury, we subjected *Acer3*^ΔHep^ and their control mice to liver I/R. In *Acer3*^ΔHep^ mice, the I/R-induced upregulation of *Acer3* expression was significantly reduced, and hepatic I/R injury was alleviated (Supplemental Figure 3). These results underscore the critical role of hepatocyte-specific *Acer3* in driving the pathogenesis of liver I/R injury. While our study focuses on hepatocyte-specific *ACER3*, the contribution of *ACER3* in other cells, such as Kupffer cells and endothelial cells, where ACER3 is highly expressed, remains to be elucidated in future investigations. These additional studies will be essential to fully understand the complex cellular interactions and the potential for ACER3-targeted therapies that could address liver injury from multiple angles.

Early studies defined CERs as a broad category of detrimental lipids due to their cell death–promoting and proinflammatory effects (21, 22). Consequently, the accumulation of total CER from sphingomyelin degradation was found to exaggerate liver I/R injury by promoting cell death and inflammation (14, 15, 18, 39). Currently, increasing attention is being paid to the fact that the bioactive functions of CER can vary depending on the acyl chains and their metabolizing enzymes, and emerging studies have uncovered the protective functions of specific CERs (12). For instance, very-long-chain CERs are known to maintain physiologic homeostasis in the liver (40), and long-chain CERs are found to support cell survival (41). Saturated CERs, including CER(d18:1/16:0), CER(d18:1/18:0), and CER(d18:1/24:0), are abundant CER species and generally have adverse pathophysiologic effects, exacerbating conditions such as insulin resistance, fatty liver, and mitochondrial fragmentation (42). ULCCs are a minor group of CERs; little is known about their pathophysiologic functions. Our previous study found that ULCC inhibits oxidative stress induced by saturated fatty acids to attenuate steatohepatitis (25). Consistently, this study demonstrated that CER(d18:1/18:1) suppresses the I/R-induced oxidative stress and attenuates liver injury. These findings unravel the hepatoprotective functions of ULCCs, and emphasize the importance of studying the specific functions of different CERs, instead of treating CERs as a single category of detrimental lipids.

A key and in-depth finding of our study is that CER(d18:1/18:1) interacts with NDUFA6, a subunit of RC-I, to inhibit RC-I activity and reduce I/R-induced ROS production (Figures 4–7). NDUFA6 is a crucial protein subunit of RC-I that maintains the normal activity and structural integrity of RC-I (9, 33). Recent advances in protein structure analysis have elucidated the structure and function of NDUFA6. NDUFA6 interacts with the transmembrane helix loop of the ND3 subunit of RC-I, stabilizing its ability to capture coenzyme Q (CoQ), which is essential for the CoQ reduction reaction (33). Mutations in NDUFA6 directly impair the catalytic activity of RC-I for CoQ reduction (43). Pioglitazone was found to interact with subunits like NDUFA6, disrupting RC-I assembly and leading to its inactivation, indicating that NDUFA6 interaction plays a critical role in maintaining RC-I functional integrity (44). NDUFA6 consists of a hydrophobic pocket that binds 4′-phosphopantetheine to anchor acyl carrier proteins, forming a subdomain crucial for RC-I activity (9, 33). We found that CER(d18:1/18:1) interacts with NDUFA6 within its hydrophobic pocket (Figure 3F and Supplemental Figure 3C). This interaction likely blocks the interaction of NDUFA6 and acyl carrier proteins to disturb the assembly of RC-I, resulting in decreased activity of RC-I (44). Regarding the functional aspect, RC-I was recently found to be dispensable for homeostasis of the adult mouse liver; loss of RC-I function displays no overt liver pathology (45, 46). In line with these findings, *Acer3* ablation per se slightly increases hepatic CER(d18:1/18:1), which binds to Ndufa6 to inhibit RC-I activity but does not cause liver dysfunction under normal conditions (25, 30), suggesting inhibition of RC-I by CER(d18:1/18:1) per se without pathologic stress, such as I/R, is not sufficient to alter liver pathology. Our data consolidate the notion that RC-I in the liver may play a more important role in stress conditions (45, 46). Interestingly, our study also revealed that proteins Atp5j, Cox6c, and Uqcrfs1, captured alongside Ndufa6 on the CER(d18:1/18:1)-immobilized chip, are functionally associated and involved in oxidative phosphorylation and electron transport chain pathways (Supplemental Figure 5, F and G), suggesting a broader role of the CER(d18:1/18:1)-NDUFA6 interaction in regulating these pathways. Future research should assess how ACER3 and CER(d18:1/18:1) affect overall mitochondrial function, including ATP production and electron transport efficiency, to provide a comprehensive understanding of their roles in mitochondrial pathophysiology in response to stress conditions.

To consolidate the findings that CER(d18:1/18:1) interacts with mitochondrial proteins in MT, we found that *Acer3* ablation not only increased CER(d18:1/18:1) in the ER but also MT and MAMs (Figure 5, C–E). MAMs serve as critical sites for lipid transport between the ER and MT (47, 48). CERs are primarily synthesized in the ER (49), and MAMs host various CER-metabolizing enzymes (23, 48, 50, 51). Additionally, CER-metabolizing enzymes like DEGS1 and CERS6 in the MAM play essential roles in regulating mitochondrial stability and biogenesis (23, 50). Previous studies have demonstrated that CER generated in the MAM can be transported to MT likely by apposing membranes, lipid vesicles, or lipid transport proteins (23, 48, 50, 51). Based on this understanding, CER(d18:1/18:1) synthesized in the ER can be transported to MT via MAMs, because MAMs act as the central hub for lipid transfer. Since *Acer3* ablation still led to an increase in CER(d18:1/18:1) in MT, ACER3 itself is unlikely to function as a direct transporter of CER(d18:1/18:1) within the ER/MAM/MT axis. Notably, ACER3, which is known to be expressed in the ER (52), was detected in the MAM fraction, indicating ACER3/Acer3 is located in MAMs (Figure 5B). Moreover, *Acer3* ablation increased CER(d18:1/18:1) levels in the ER and MAMs under basal conditions and I/R conditions (Supplemental Figure 6, A–D). These findings suggest that ACER3 could control the levels of CER(d18:1/18:1) by catalyzing its degradation within both the ER and MAM, which in turn influences CER(d18:1/18:1) transport from the ER to MT via MAMs. Alternatively, CER(d18:1/18:1) transfer to MT could bypass the MAM. For instance, lipid transfer proteins, such as members of the STARD family, may shuttle CER(d18:1/18:1) between the ER and MT (53, 54). Vesicular transport may also contribute, with CER(d18:1/18:1) encapsulated in vesicles that bud from the ER and deliver their content to MT through vesicle fusion (55). These findings provide insights into the regulatory role of ACER3 in CER(d18:1/18:1) trafficking within the ER/MAM/MT axis, warranting further investigation into its specific contributions to mitochondrial functions.

Oxidative stress is driven by ROS production that induces mitochondrial dysfunction and damage to initiate liver I/R injury (3, 4). Adding to our previous findings showing that *Acer3* ablation increases CER(d18:1/18:1) and prevents fatty-acid-induced oxidative stress (25), our present study demonstrated that *Acer3* ablation increases CER(d18:1/18:1) to inhibit ROS production and oxidative stress in the liver with I/R injury (Figures 5 and 6). RC-I plays a critical role in producing ROS to drive I/R injury (5–8). I/R increases transmembrane potential and accumulates CoQH_2_ to induce reverse electron transfer from CoQH_2_ to NAD^+^, reducing the NAD^+^/NADH ratio. This causes RC-I to release electrons and generate significant ROS, which initiates and drives the progression of I/R injury (56, 57). Studies have shown that inhibiting RC-I activity through agents such as rotenone, *S*-nitrosating agents, and metformin reduces ROS production and alleviates I/R injury (4, 7, 8, 11). Recent research also indicates that mutations in RC-I subunits can prevent RC-I–mediated ROS generation, further mitigating I/R injury (8). According to these findings, we considered that *Acer3* ablation reduces ROS production primarily by promoting the CER(d18:1/18:1)-Ndufa6 interaction, which inhibits RC-I activity in the liver with I/R injury. Notably, *Ndufa6* knockdown weakens the suppression of ROS production and oxidative stress in the I/R-injured liver of *Acer3*-deficient and CER(d18:1/18:1)-treated mice (Figure 7), underscoring the crucial role of Ndufa6 in mediating the protective effects of CER(d18:1/18:1) against I/R-induced oxidative stress. These findings extend the molecular insights into how individual CER regulates oxidative stress and highlight NDUFA6 as a potentially druggable subunit of RC-I for liver I/R injury.

### Conclusion.

In conclusion, our study elucidates how ACER3 and its CER(d18:1/18:1) substrate regulate oxidative stress and liver I/R injury. By uncovering the interaction between CER(d18:1/18:1) and RC-I subunit NDUFA6, we provide molecular insights into the pathogenesis of liver I/R injury. These findings suggest that targeting the CER(d18:1/18:1)-NDUFA6 interaction could be a promising therapeutic strategy for mitigating oxidative stress and protecting the liver against I/R injury.

## Methods

Further details can be found in the Supplemental Methods.

### Sex as a biological variable.

Our study considered sex as a biological variable in *Acer3*^+/+^ and *Acer3^–/–^* mice subjected to IRI. Our findings are expected to be relevant for both sexes.

### Patient samples.

Clinical normal liver specimens were collected from regions distant from (>5 cm) liver lesions in patients undergoing partial hepatectomy with or without the PM at the Division of Hepatobiliopancreatic Surgery, Department of General Surgery, Nanfang Hospital, Southern Medical University from April 2019 to April 2021. The PM was performed intermittently using a Foley tube to encircle the porta hepatis for bleeding control during liver parenchyma transection, resulting in liver I/R injury as an adverse effect (26). A total of 39 patients were included: 22 patients without PM (non-I/R group) and 17 patients with PM-induced liver injury (I/R group). The patients’ demographic and clinical information are provided in Supplemental Table 1.

### Animal models.

All mice were bred and reared under specific-pathogen-free (SPF) conditions with a 12 hour/12 hour light/dark cycle at 21°C and 50%–55% humidity at the animal facilities of Southern Medical University. *Acer3^–/–^* and wild-type littermate controls (*Acer3*^+/+^) with a C57BL/6J genetic background were generated as in our previous study (30). *Acer3*^ΔHep^ mice and littermate controls (*Acer3^fl/fl^*) were generated by CRISPR/Cas-mediated genome engineering (Cyagen Biosciences Inc). Exons 3 and 4 of the *Acer3* gene were selected as the conditional KO (cKO) target regions. Homologous arms and the cKO region were amplified via PCR to construct the targeting vector. The guide RNA (gRNA) targeting exons 3 and 4, along with the donor vector containing *loxP* sites, and Cas9 mRNA were coinjected into fertilized mouse eggs to generate the F0 generation of cKO mice. The F0 mice were then crossed with mice expressing hepatocyte-specific Cre recombinase driven by the albumin (*Alb*) gene promoter to produce the F1 generation mice. The F1 heterozygous mice were subsequently bred to generate homozygous *Acer3*^ΔHep^ and *Acer3^fl/fl^* mice. The identifications of genotypes are shown in Supplemental Figure 4. CER(d18:1/18:1) treatment in mice was performed as previously described (58). Aged-matched C57BL/6J wild-type male mice were intraperitoneally injected with 5 mg/kg/day CER(d18:1/18:1) (Avanti Polar Lipids) dissolved in 0.5% sodium carboxymethylcellulose (CMC-Na, Sigma-Aldrich), and control mice were injected with 0.5% CMC-Na. The CER(d18:1/18:1) treatment was performed once every day starting 3 days before surgery and once after surgery. *Ndufa6* knockdown in 6- to 8-week-old *Acer3^–/–^* male mice was performed using liver-directed type 8 adeno-associated viruses (AAV) (3 × 10^11^ PFU) carrying shRNA targeting the *Ndufa6* gene (shNdufa6) via tail vein injection. Corresponding control shRNA (shCON) with green fluorescent protein (GFP) (Obio Biology) was used for control mice. The targeted sequences of vectors used for knocking down *Ndufa6* are listed in Supplemental Table 2. The I/R operation was performed 3 weeks after the AAV injection with confirmed *Ndufa6* knockdown. Mice 8–10 weeks old were used in this study. Warm liver I/R injury was induced by clamping the porta hepatis for 1 hour, followed by reperfusion for 6 or 24 hours. Mice were sacrificed at indicated time points and liver tissues and serum were collected for experiments.

### Statistics.

Data are expressed as mean ± SD. Statistical significance was determined using 2-tailed Student’s *t* test or 1-way ANOVA followed by Tukey’s post hoc test for multiple comparisons. A *P* value of less than 0.05 was considered statistically significant.

### Study approval.

All procedures involving human samples were approved by the Experimentation Ethics Committee of Nanfang Hospital, Southern Medical University (no. NFEC-2019-029) and written informed consent was obtained from all patients. All animal experiments were approved by the Animal Care and Use Committee of Nanfang Hospital Southern Medical University.

### Data availability.

All data are available in this manuscript and in the Supporting Data Values file. The data supporting the findings of this study are available from the corresponding authors upon reasonable request.

## Author contributions

KW and C Li conceived and designed the study. KW, L Liao, HL, PH, QL, BZ, CX, XM, XD, C Liu, and JL performed the experiments, data collection, and statistical analysis. KW, L Liao, Y Lei, MZ, CM, RX, XW, JZ, BW, and Y Li analyzed the data and wrote the manuscript. C Li, KW, BW, and Y Li contributed to data interpretation and critical revision of the manuscript. All authors reviewed and approved the final version of the manuscript. KW, LL, HL, and PH are co–first authors; KW is listed first based on his leading contributions to the project’s conceptualization and experimental design, execution of key experiments, and pivotal role in data analysis, interpretation, and writing the manuscript.

## Supplementary Material

Supplemental data

Unedited blot and gel images

Supporting data values

## Version 1 04/17/2025

In-Press Preview

## Version 2 05/22/2025

Electronic publication
