# Supplementary material for: Ceramide(d18:1/18:1)-NDUFA6 interaction inactivates respiratory complex I to attenuate oxidative-stress-driven pathogenesis in liver ischemia/reperfusion injury
Source: JCI Insight. 2025 Apr 17;10(10):e187083. doi: 10.1172/jci.insight.187083 (PMC12128967; doi:10.1172/jci.insight.187083)
Supplement: Supplemental data [file jciinsight-10-187083-s126.pdf]

Supplemental Methods

Liver *in situ* hybridization (ISH)

Human and mouse liver tissue sections were subjected to ISH using RNA scope ISH kits and probes (Advanced Cell Diagnostics, CA, USA) as described [1]. Liver sections were pretreated by repair reagents and then hybridized with the specific oligonucleotide probe targeting the region of the *ACER3*/*Acer3* gene. After amplification of the staining signal, samples were hybridized with a probe labeled with horseradish peroxidase (HRP). Positive staining was detected with a red color. Each RNA transcript exhibited a distinct dot or cluster of signals.

Measurement of serum aminotransferases

Mouse serum alanine aminotransferase (ALT) and aspartate aminotransferase (AST) levels were measured using a commercial kit (Sigma-Aldrich, MO, USA).

Evaluation of Reactive Oxygen Species (ROS) production

H_2O_2 levels in liver tissues were measured using the Amplex Red Hydrogen Peroxide/Peroxidase Assay Kit (Thermo Fisher Scientific, Waltham, MA). The absorbance of samples and standards at 560 nm was measured using the Multifunctional Microplate Reader (Molecular Devices, San Jose, CA, USA), and the concentration of H_2O_2 in the samples was calculated based on the standard curve. ROS detection was performed using MitoSOX Red/dihydroethidium (DHE) probes on frozen liver sections as described [2]. Images were captured using the Intelligently Designed Microscope (Olympus, Shinjuku-ku, Tokyo, Japan), and fluorescence intensity from at least five randomly selected areas (magnification, 20 ×) were quantified and analyzed using Image J software (NIH, Bethesda, MD).

Histological Analysis

Liver tissues were fixed in 10% formalin, embedded in paraffin, sectioned, and stained with hematoxylin and eosin (H&E) for histopathological evaluation. Necrotic

areas were quantified using Image J software. Immunohistochemistry (IHC) staining was performed using a VECTASTAIN® Elite® ABC Kit (Rabbit IgG) (VECTOR, Burlingame, CA, USA) and 3,3’-diaminobenzidine (DAB) Peroxidase Substrate Kit (VECTOR, Burlingame, CA, USA) following the manufacturer's instructions. Liver sections were subjected to IHC staining with antibodies against LY6G (Abcam, Cambridge, MA, USA). Representative pictures were taken using the Intelligently Designed Microscope.

Immunoprecipitation (IP)

The FLAG-tagged NDUFA6 coding sequence was constructed from the respective cDNA clones using 3 × FLAG-tag-encoding oligonucleotides, followed by insertion into the pcDNA$^{TM3.1}$ vector (Thermo Fisher Scientific, Waltham, MA, USA). The human-liver-derived cell lines HepG2 was obtained from the Shanghai Cell Bank of the Academy of Chinese Sciences and Liver Cancer Institute (Zhongshan Hospital, Fudan University, China). HepG2 cells were transfected with plasmids containing FLAG-tagged NDUFA6 or empty vectors. After 24-hour transfection, the cells were treated with CER(d18:1/18:1) for another 24 hours. The proteins were extracted using a detergent-free Minute^{TM} Total Protein Extraction Kit (Invent Biotechnologies, Inc., Eden Prairie, USA). The expression efficiency of FLAG and NDUFA6 protein was verified by Western blot. EZview Red Anti-FLAG M2 Affinity Gel (Sigma-Aldrich, MO, USA) was washed with Tris Buffered Saline solution (50 mM Tris HCl, 150 mM NaCl, pH 7.4) twice. Diluted protein lysates (100 ul) were incubated with EZview Red Anti-FLAG M2 Affinity Gel (20 ul) and shaken slowly at 4℃ overnight. The next day, the samples were centrifuged at 8200 g for 30 s at 4 ℃ and the supernatant was removed. The precipitates were gently mixed and incubated with Tris-buffered saline solution for 5 min, centrifuged at 8200 g for 30 s, and the supernatant was removed. Repeat the washing step three times. The above precipitations were incubated with 150 μl FLAG peptide (Sigma-Aldrich, MO, USA) at 4 ℃ for 30 min. The suspension was centrifuged at 4 ℃, 8200 g for 30 s. The supernatants were verified for the efficiency of IP and then subjected to ceramide (CER) extraction for

CER measurement.

RNA isolation and quantitative real-time polymerase chain reaction (RT-qPCR) analysis

Total RNA was extracted from mouse liver tissues using TriZol reagent (Invitrogen, Waltham, MA, USA). Subsequently, mRNA was transcribed into cDNA by 5 × PrimeScript RT Master Mix (TaKaRa, Kusatsu, Shiga, Japan). RT-qPCR analyses were done on LightCycler 480 (Roche, Auckland, New Zealand). Relative gene expression levels were determined using ΔΔCT calculation, and mRNA levels were relative to the control condition where indicated. *Actb* was used as the housekeeping control. Primer's sequences of indicated mRNA of human and mouse genes employed for the RT-qPCR are illustrated in Table S4.

Western blotting

Liver tissues and cell pellets were homogenized in RIPA buffer (Thermo Scientific, Waltham, MA, USA) to extract whole-cell protein. The concentrations of protein extracts were determined using a bicinchoninic acid (BCA) protein determination kit (Thermo Fisher Scientific, Waltham, MA, USA). An equal amount of denatured protein with loading buffer was loaded in each lane of SDS-PAGE tris-glycine gels and transferred onto polyvinylidene fluoride (PVDF) membranes (Roche, Auckland, New Zealand) using an electrophoretic wet Western blot transfer system. After blocking, the membranes were incubated with primary antibodies overnight followed by incubation with HRP-conjugated secondary antibodies. The signals were visualized using the ECL Prime Western Blotting Detection Reagent (Cytiva, Westboro, USA). Actb was used as the loading control. The information on the used antibodies is listed in Table S5.

Lipid Extraction and CER measurements

The measurement of CER and CER metabolites was performed following to protocol reported by Zhihong Jiang [3]. In brief, 225 μl MeOH was added to the homogenates of

liver tissues and HepG2 cells, as well as the supernatants of IP. After vortexing, 50 µl IS cocktail and 750 µl pre-cooling alkaline methyl tert-butyl ether (MTBE) were added. The mixture was incubated in a Thermomixer Comfort at 650 rpm for 1 hour at 4 °C. Afterward, 188 µL MilliQ water was added, and the samples were centrifuged at 10,000 g for 10 min at 4 °C. The upper organic layer (600 µl) was transferred to a new tube and dried under a continuous stream of nitrogen to obtain lipid extracts (1L/min N_2 at 25 °C). After removing the middle layer, the lower layer was added with 903 µl MeOH and stored at -80 °C for 4 hours to precipitate protein. Protein pellets were collected after centrifugation at 19,803 g for 30 min at 4 °C and resuspended in buffer solution (1% SDS, 150 mM NaCl, 50 mM Tris, pH 7.8) for protein quantification by BCA. The dried powders from the upper organic layer were resuspended in 100 µl 30% mobile phase B (IPA/ACN, 9/1 (v/v), 0.1 % formic acid, 10 mM ammonium formate, and 5 µM phosphoric acid). CERs were determined by LC-MS/MS performed on prelude SPLC coupled with TSQ Quantiva system (Thermo Fisher Scientific, Waltham, MA, USA). The internal standard cocktail contained CER(d18:1/17:0), sphingosine (SPH) (d17:1), sphingosine-1-Phosphate (S1P) (d17:1). CER standards were purchased from Avanti Polar Lipids (Birmingham, Alabama, USA), including CER(d18:1/6:0), CER(d18:1/16:0), CER(d18:1/18:0), CER(d18:1/18:1), CER(d18:1/20:0), CER(d18:1/22:0), CER(d18:1/24:0), CER(d18:1/24:1), SPH(d18:1), and S1P(d18:1). Amounts of sphingolipids were quantified using standard curves and normalized to protein contents.

CER(d18:1/18:1)-Targeted Protein Fishing by Surface Plasmon Resonance (SPR)

Proteins that potentially interacted with CER(d18:1/18:1) were caught by SPR and identified by proteomics. Three 3D PCL chips were prepared: positive, negative, and blank. Each chip was coated with 20 µl of 2.5 mM CER(d18:1/18:1) (methanol for the blank) and dried at 37°C for 3 hours. After UV cross-linking at 365 nm for 15 minutes, the chips were washed with dimethylformamide (DMF, Sigma-Aldrich, MO, USA), ethanol, and water, then dried with N2. Liver tissue proteins were extracted, diluted to 0.625 µg/µl, and divided into positive, negative, and blank groups. The

protein suspension of the negative group was incubated with 2.5 mM CER(d18:1/18:1) at 4°C for 2 hours, heated at 60°C for 1 hour, and centrifuged. The samples were then incubated with the corresponding chips to fish for proteins. After incubation, the bound proteins were eluted with glycine-HCl and prepared for proteomics analysis using FASP. Proteins were identified via EASY-nLC1200-UPLC and analyzed against the UniProt database. Specific interactors were identified by subtracting proteins found in negative and blank controls from those in the positive group. Molecular docking was performed using AutoDock Vina and analyzed with PyMOL.

Protein-Lipid Docking

The structure of CER(d18:1/18:1) (Compound CID: 5283563) was downloaded from the PubChem database (https://pubchem.ncbi.nlm.nih.gov/). The crystal structures of mouse proteins that potentially interacted with CER(d18:1/18:1) were obtained from the RCSB Protein Data Bank (http://www.rcsb.org/) or AlphaFold Protein Structure Database (https://alphafold.com/) (Table S3). All redundant atoms except the chain involved in docking were deleted using Pymol (version 2.3.0). AutoDock Vina (version 1.1.2) was used to simulate docking between CER(d18:1/18:1) and these proteins [4]. The interaction affinities were analyzed using Pymol and Ligplot (version 2.2.4). Ndufa6 with the highest predicted affinity was selected for further docking analysis using human NDUFA6 protein structures. The crystal structures of human NDUFA6 protein (UniProt ID: 5xtb, 5xth, 5xti, and 5xtd) were downloaded from the RCSB Protein Data Bank (http://www.rcsb.org/). All redundant atoms except the chain involved in docking were deleted. The protein structure was treated in several steps including residue repair, protonation, and partial charges assignment in the AMBER ff14SB force field. The DMS tool was employed to build the molecular surface of the receptor using a probe atom with a 1.4 Å radius. The binding pocket was defined by the crystal ligand and spheres were generated filling the site by employing the Sphgen module in UCSF Chimera [5]. Subsequently, the DOCK 6.9 [6] (https://www.cgl.ucsf.edu) program was utilized to execute semi-flexible docking where 10000 different orientations were produced. Clustering analysis was performed

(RMSD threshold 2.0 Å) for candidate poses, and the best-scored molecular modeling was output. The complex of CER(d18:1/18:1) and human NDUFA6 protein (PDB code 5xtb) were illustrated. Discovery Studio software (Omaha, Nebraska, USA) was used to analyze the interaction.

Subcellular Fractionation

The isolation of endoplasmic reticulum (ER), mitochondria-associated membranes (MAM), and mitochondria (MT) was performed as described [7]. In brief, fresh liver tissues were collected from mice and washed with buffer (225mM mannitol, 75mM sucrose, 0.5% BSA, 0.5mM EGTA, and 30mM Tris-HCl pH 7.4). The tissues were homogenized, and the homogenate was centrifuged at 740 g for 10 minutes at 4°C to remove debris. The supernatant was centrifuged at 9000 g for 10 minutes to pellet crude mitochondria, which were then resuspended in buffer (250mM mannitol, 5mM HEPES (pH 7.4), and 0.5mM EGTA). The crude mitochondria were further purified using Percoll density gradient centrifugation at 95000 g for 30 minutes at 4°C. The dense white band at the bottom contained purified mitochondria, while the diffused band above contained MAM. The supernatant from the initial centrifugation was further centrifuged at 100000 g for 60 minutes to isolate ER. ER, MT, and MAM fractions were confirmed by immunoblotting with markers for each compartment: Carl for ER, Cytc and Vdac for MT, and Facl4 for MAM. CERs, SPH, and S1P were extracted from MT, and MAM fractions and measured and normalized as mentioned above.

Respiratory Complex I (RC-I) Activity Assay

The activity of RC-I was measured using the Mitochondrial Complex I Activity Assay Kit MAK359 (Sigma-Aldrich, MO, USA), which primarily detects the reduction activity of CoQ. The procedure was performed according to the manufacturer's instructions. MT was isolated from liver tissues. The assay uses decylubiquinone (a CoQ analog) as the electron acceptor, which is reduced to decylubiquinol by RC-I. Equal protein amounts of MT were used for assays. The RC-I dye in its oxidized form

absorbs light at 600 nm (A600). As the dye accepts electrons from decylubiquinol, it is reduced, resulting in a decrease in A600 absorbance. The change in absorbance (A600) is recorded to determine RC-I activity colorimetrically.

Statistical Analysis

Data are expressed as mean ± SD. Statistical significance was determined using 2-tailed Student's t-test or one-way ANOVA followed by Tukey's post hoc test for multiple comparisons. A p-value < 0.05 was considered statistically significant.

References

1. Wang, F. et al. RNAscope: a novel in situ RNA analysis platform for formalin-fixed, paraffin-embedded tissues. *J. Mol. Diagn.* 14, 22-29 (2012).
2. Wang, Q. & Zou, M. H. Measurement of Reactive Oxygen Species (ROS) and Mitochondrial ROS in AMPK Knockout Mice Blood Vessels. *Methods Mol Biol.* 1732, 507-517 (2018).
3. Wang, J. R. et al. Improved sphingolipidomic approach based on ultra-high performance liquid chromatography and multiple mass spectrometries with application to cellular neurotoxicity. *Anal. Chem.* 86, 5688-5696 (2014).
4. Eberhardt, J., Santos-Martins, D., Tillack, A. F. & Forli, S. AutoDock Vina 1.2.0: New Docking Methods, Expanded Force Field, and Python Bindings. *J. Chem Inf. Model.* 61, 3891-3898 (2021).
5. Huang, C. C., Meng, E. C., Morris, J. H., Pettersen, E. F. & Ferrin, T. E. Enhancing UCSF Chimera through web services. *Nucleic Acids Res.* 42, W478-W484 (2014).
6. Allen, W. J. et al. DOCK 6: Impact of new features and current docking performance. *J. Comput. Chem.* 36, 1132-1156 (2015).
7. Wieckowski, M. R., Giorgi, C., Lebiedzinska, M., Duszynski, J. & Pinton, P. Isolation of mitochondria-associated membranes and mitochondria from animal tissues and cells. *Nat. Protoc.* 4, 1582-1590 (2009).

Supplemental figures and tables

Figures S1–S6 and Tables S1-S5

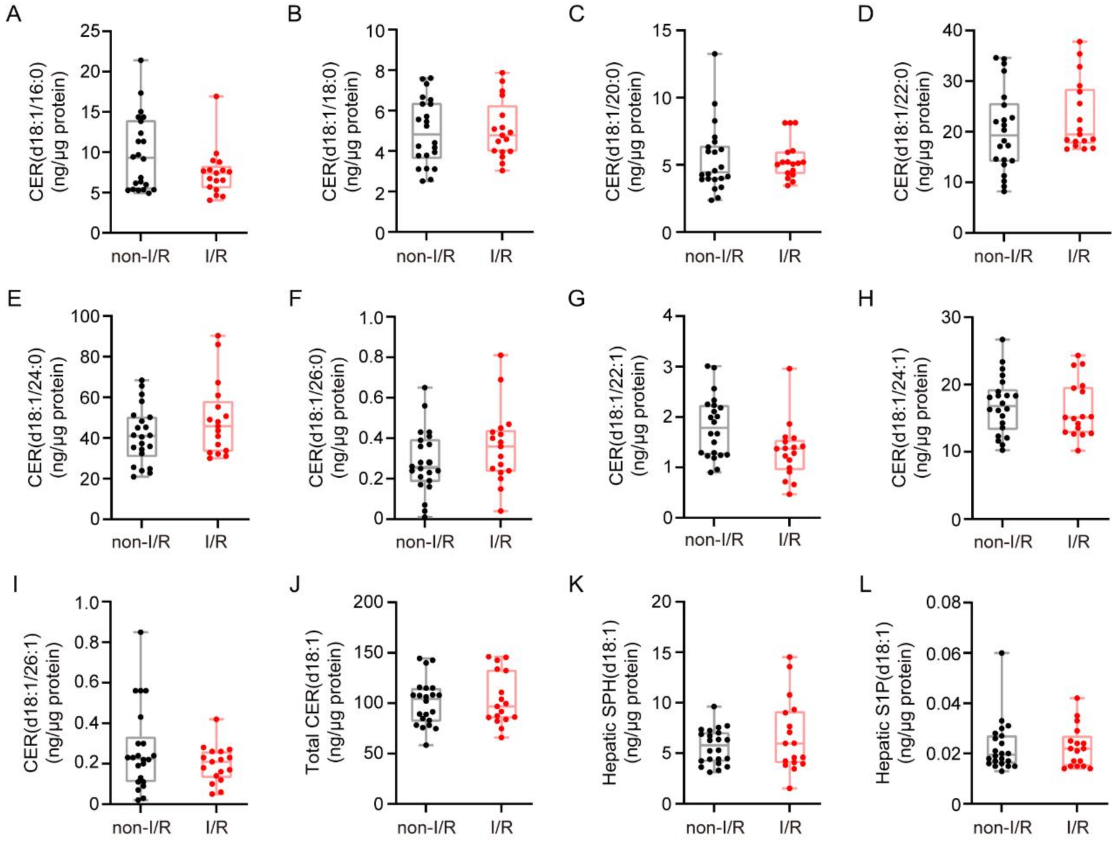

Supplementary Figure 1. CER, SPH, and S1P levels in liver tissues from patients with and without I/R injury.

(**A-J**) Levels of measured CER species in resected liver tissues from non-I/R and I/R groups, including CER(d18:1/16:0) (**A**), CER(d18:1/18:0) (**B**), CER(d18:1/20:0) (**C**), CER(d18:1/22:0) (**D**), CER(d18:1/24:0) (**E**), CER(d18:1/26:0) (**F**), CER(d18:1/22:1) (**G**), CER(d18:1/24:1) (**H**), CER(d18:1/26:1) (**I**), and Total CER(d18:1) (**J**).

(**K and L**) Hepatic levels of SPH(d18:1) (**K**) and S1P(d18:1) (**L**) in liver tissues from non-I/R and I/R groups.

Data in **A-L**, n = 22 in the non-I/R group, and 17 in the I/R group. Statistical

significance was determined using 2-tailed Student's t-test. * $P < 0.05$, ** $P < 0.01$, *** $P < 0.001$.

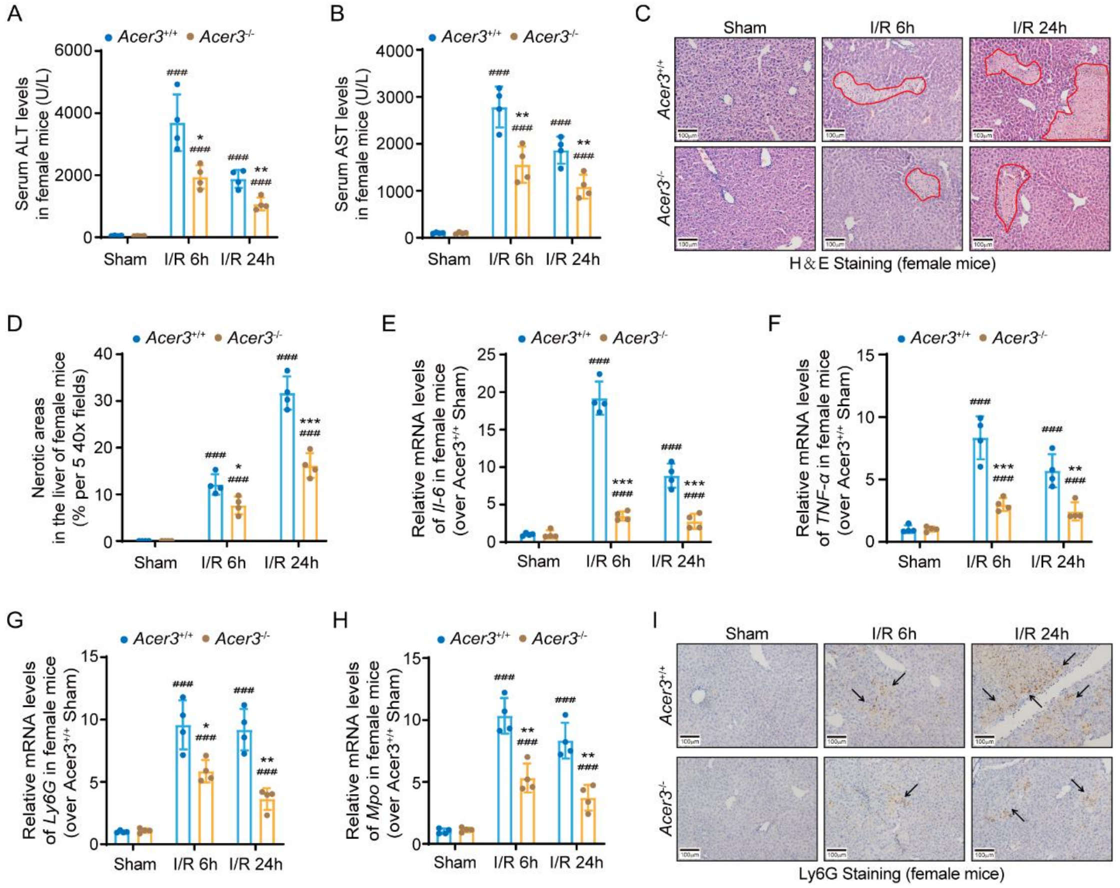

Supplementary Figure 2. Gobal *Acer3* ablation attenuates liver I/R injury in female mice.

(**A** and **B**) Serum ALT (**A**) and AST (**B**) levels in *Acer3*$^{+/+}$ and *Acer3*$^{-/-}$ female mice under sham operation and at 6 hours and 24 hours post-I/R.

(**C** and **D**) Representative H&E staining of liver sections with circle areas indicating necrotic foci (**C**) and quantification of necrotic area (**D**) in *Acer3*$^{+/+}$ and *Acer3*$^{-/-}$ female mice under sham operation and at 6 hours and 24 hours post-I/R.

(**E-I**) Relative mRNA levels of pro-inflammatory cytokines *Il-6* (**E**) and *Tnf-α* (**F**) and neutrophil markers *Ly6g* (**G**) and *Mpo* (**H**) in liver tissues from *Acer3*$^{+/+}$ and *Acer3*$^{-/-}$ female mice under sham operation and at 6 hours and 24 hours post-I/R. Representative Ly6G staining of liver sections with black arrows indicating inflammatory infiltration under sham operation and at 6 hours and 24 hours post-I/R (**I**).

Images in **C** and **I** represent the results of four pairs of mice per group. Data in **A**, **B**, and **D**-**H** are expressed as mean ± SD, n = 4 per group. Statistical significance was determined using one-way ANOVA followed by Tukey's test for multiple comparisons. # in **A**, **B**, and **D-H** indicate comparisons between mice with Sham and I/R. $^{*/\#}P < 0.05$, $^{**/\#\#}P < 0.01$, $^{***/\#\#\#}P < 0.001$.

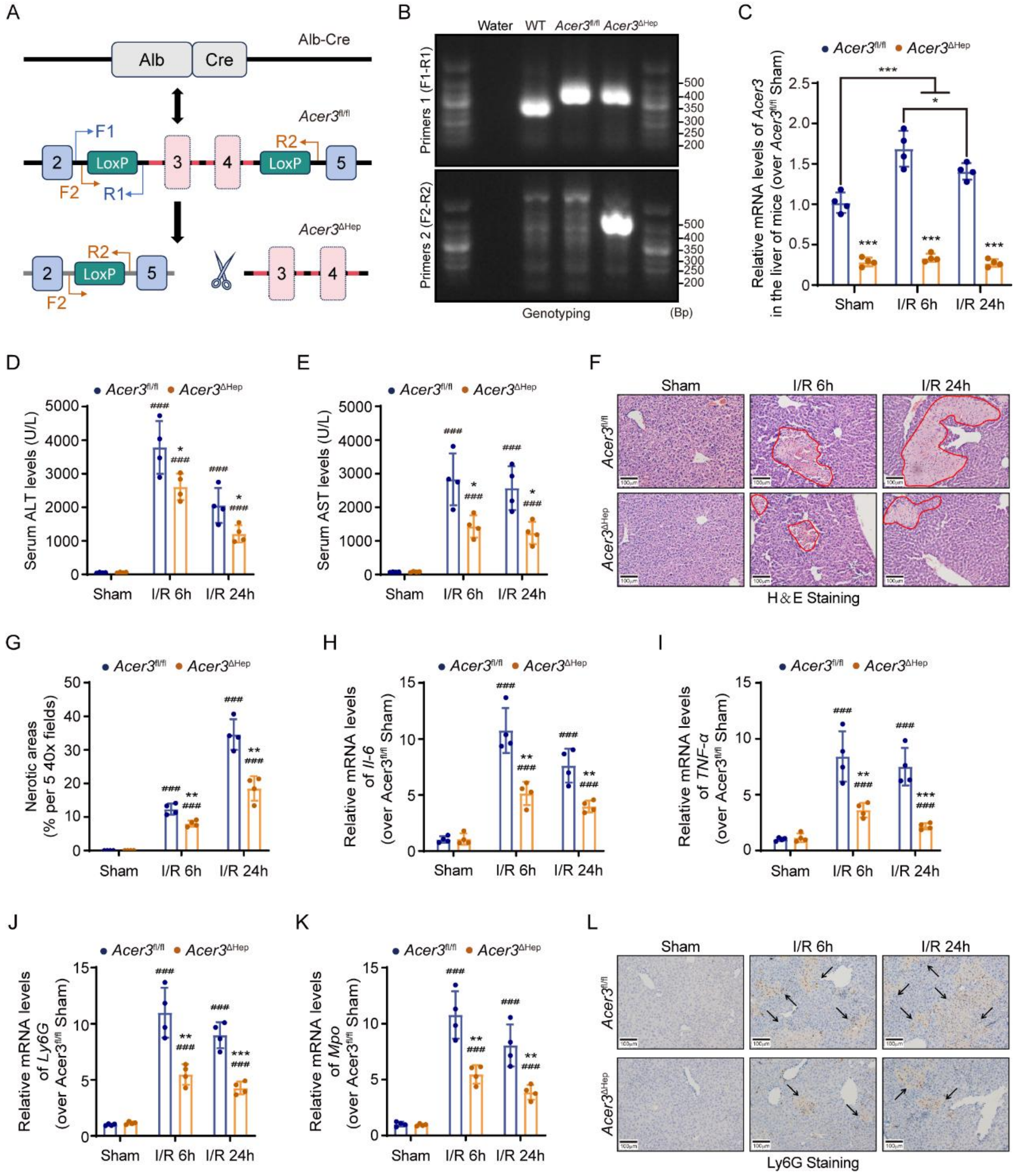

Supplementary Figure 3. Establishment of hepatocyte-specific *Acer3* deficient mice and hepatocyte-specific *Acer3* ablation attenuates liver I/R injury in male mice.

(**A**) Schematic diagram depicting the design of *Acer3*$^{fl/fl}$ and *Acer3*$^{\Delta Hep}$ mice.

(**B**) Genotyping of mouse tail in *Acer3*$^{fl/fl}$ and *Acer3*$^{\Delta Hep}$ mice.

(**C**) The mRNA levels of *Acer3* in the liver of *Acer3*$^{fl/fl}$ and *Acer3*$^{\Delta Hep}$ male mice under sham operation and at 6 hours and 24 hours post-I/R.

(**D** and **E**) Serum ALT (**D**) and AST (**E**) levels in *Acer3*$^{fl/fl}$ and *Acer3*$^{\Delta Hep}$ male mice under sham operation and at 6 hours and 24 hours post-I/R.

(**F** and **G**) Representative H&E staining of liver sections with circle areas indicating necrotic foci (**F**) and quantification of necrotic area (**G**) in *Acer3*$^{fl/fl}$ and *Acer3*$^{\Delta Hep}$ male mice under sham operation and at 6 hours and 24 hours post-I/R.

(**H**-**L**) Relative mRNA levels of pro-inflammatory cytokines *Il-6* (**H**) and *Tnf-α* (**I**) and neutrophil markers *Ly6g* (**J**) and *Mpo* (**K**) in liver tissues from *Acer3*$^{fl/fl}$ and *Acer3*$^{\Delta Hep}$ male mice under sham operation and at 6 hours and 24 hours post-I/R. Representative Ly6G staining of liver sections with black arrows indicating inflammatory infiltration under sham operation and at 6 hours and 24 hours post-I/R (**L**).

Images in **K** and **L** represent the results of four pairs of mice per group. Data in **C**, **D**, **E**, and **G**-**K** are expressed as mean ± SD, n = 4 per group. Statistical significance was determined using one-way ANOVA followed by Tukey's test for multiple comparisons. $^{\#}$ in **D**, **E**, and **G**-**K** indicate comparisons between mice with Sham and I/R. $^{*/\#}P < 0.05$, $^{**/\#\#}P < 0.01$, $^{***/\#\#\#}P < 0.001$.

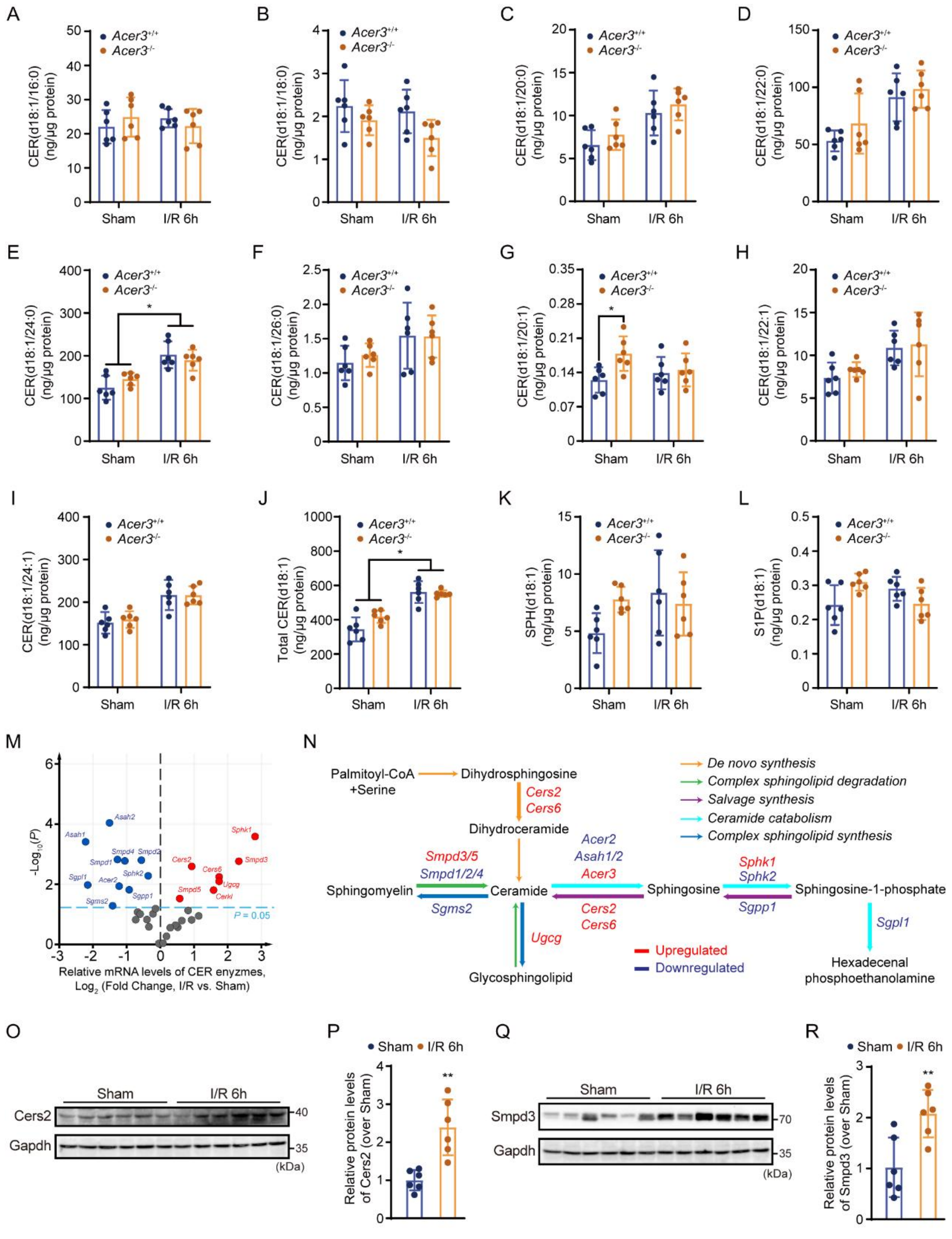

Supplementary Figure 4. CER, SPH, and S1P levels and sphingolipid metabolic enzyme expressions in liver tissues from *Acer3*$^{+/+}$ and *Acer3*$^{-/-}$ male mice.

(**A**-**L**) Levels of CER species and metabolites in liver tissues from *Acer3*$^{+/+}$ and *Acer3*$^{-/-}$ mice under sham conditions and at 6 hours post-I/R, including CER(d18:1/16:0) (**A**),

CER(d18:1/18:0) (**B**), CER(d18:1/20:0) (**C**), CER(d18:1/22:0) (**D**), CER(d18:1/24:0)

(**E**), CER(d18:1/26:0) (**F**), CER(d18:1/20:1) (**G**), CER(d18:1/22:1) (**H**), CER(d18:1/24:1) (**I**), total CER(d18:1) (**J**), SPH(d18:1) (**K**), and S1P(d18:1) (**L**).

(**M**) Volcano plot of the mRNA levels of sphingolipid metabolic enzymes in the liver of male mice under sham operation and at 6 hours post-I/R.

(**N**) Schemed diagram of dysregulation in sphingolipid metabolism in the liver of male mice at 6 hours post-I/R.

(**O-R**) The protein levels of cers2 (**O**) and smpd3 (**P**), quantification of cers2 (**Q**) and smpd3 (**R**) protein levels in the liver of male mice under sham operation and at 6 hours post-I/R.

Images in **O** and **Q** represent the results of six pairs of mice per group. Data in **A**-**M**, **P**, **and R** are expressed as mean ± SD, n = 6 per group. Data are expressed as mean ± SD, n = 6 per group. Statistical significance was determined using 2-tailed Student's *t*-test (**P** and **R**) or one-way ANOVA followed by Tukey's test for multiple comparisons (**A-L**). $*P < 0.05$, $**P < 0.01$.

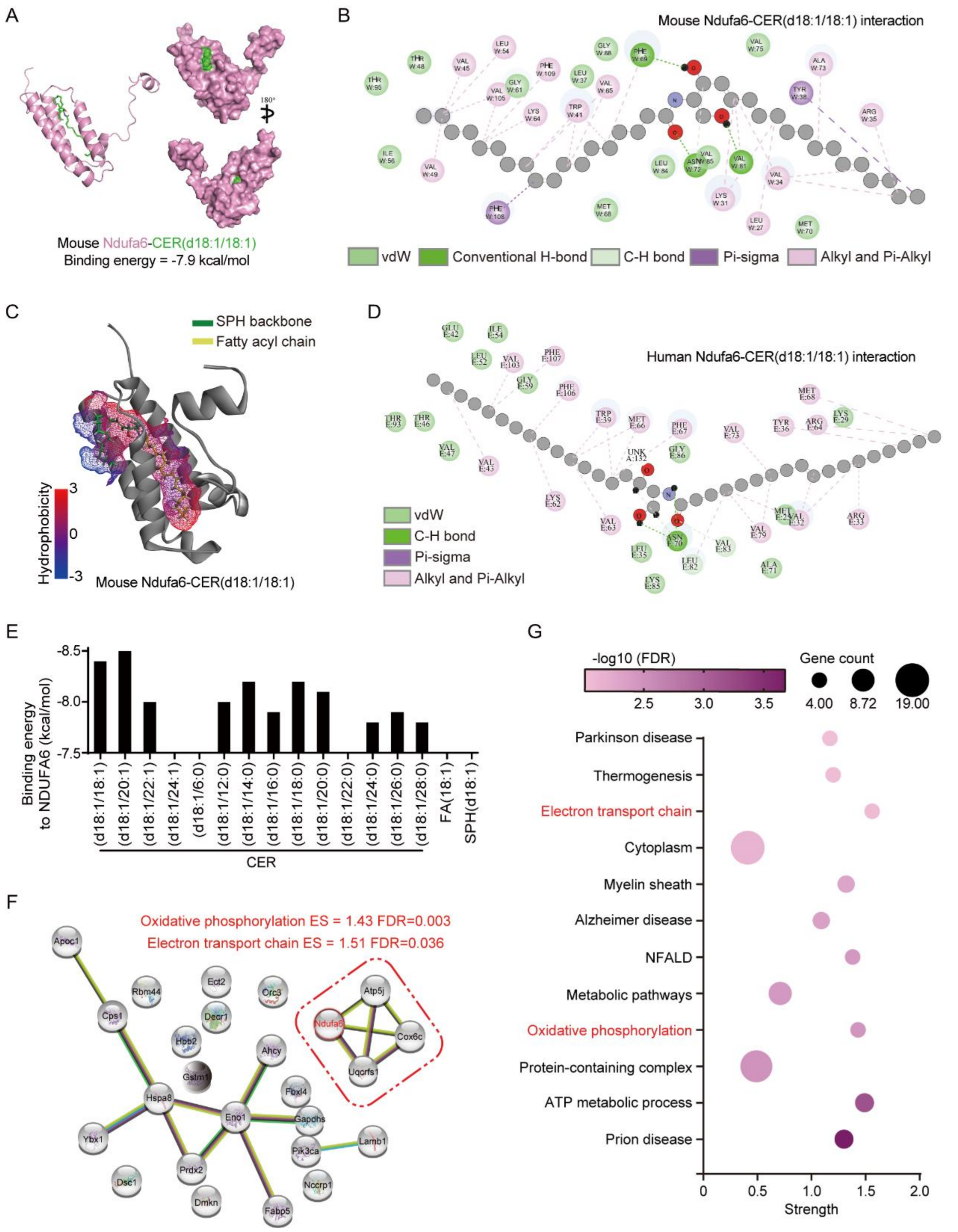

Supplementary Figure 5. Interaction between CER(d18:1/18:1) and NDUFA6 and pathway enrichment analysis.

(**A** and **B**) Molecular interaction model of mouse Ndufa6 with CER(d18:1/18:1).

(**C**) Hydrophobicity analysis of the interaction of mouse Ndufa6-CER(d18:1/18:1).

(**D**) Interaction details of human NDUFA6 and CER(d18:1/18:1).

(**E**) The predicted binding energy of CER(d18:1) species, FA(18:1), and SPH(d18:1)

to human NDUFA6 protein evaluated by AutoDock Vina software.

(**F**) Functional protein association networks analysis using STRING, showing the interaction network of NDUFA6 enriched in oxidative phosphorylation and electron transport chain pathways.

(**G**) Pathway enrichment analysis of the proteins interacting with CER(d18:1/18:1), highlighting significant pathways, including the electron transport chain and oxidative phosphorylation.

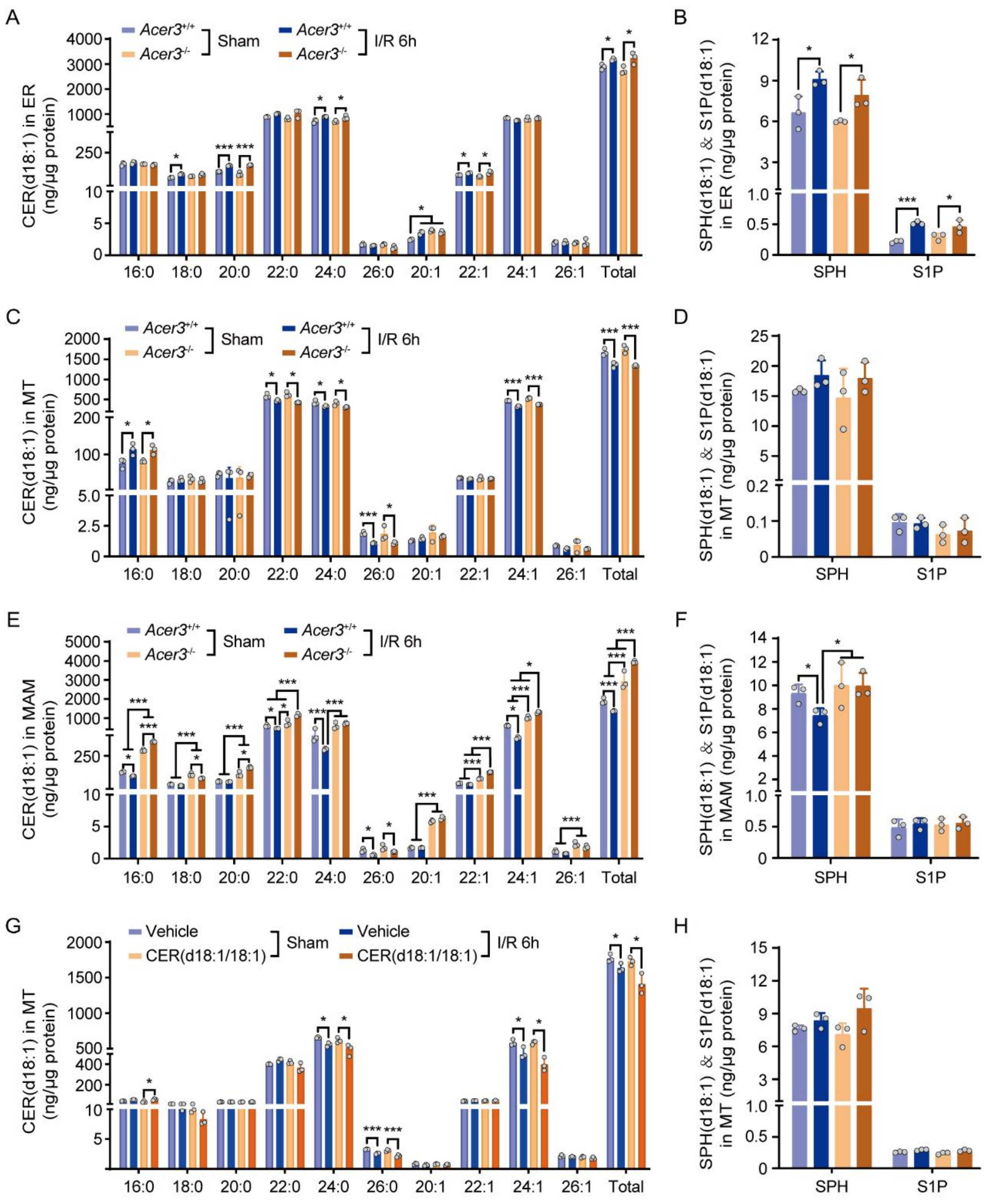

Supplementary Figure 6. CER, SPH, and S1P levels in ER, MT, and MAM fractions from the liver of *Acer3*$^{+/+}$, *Acer3*$^{-/-}$, and CER(d18:1/18:1)-treated mice.

(**A** and **B**) Levels of CER species (**A**), SPH, and S1P (**B**) in the ER fraction from *Acer3*$^{+/+}$ and *Acer3*$^{-/-}$ mice under sham operation and at 6 hours post-I/R.

(**C** and **D**) Levels of CER species (**C**), SPH, and S1P (**D**) in the MT fraction from *Acer3*$^{+/+}$ and *Acer3*$^{-/-}$ mice under sham operation and at 6 hours post-I/R.

(**E** and **F**) Levels of CER species (**E**), SPH, and S1P (**F**) in the MAM fraction from *Acer3*$^{+/+}$ and *Acer3*$^{-/-}$ mice under sham operation and at 6 hours post-I/R.

(**G** and **H**) Levels of CER species (**G**), SPH, and S1P (**H**) in the MT fraction from the vehicle and CER(d18:1/18:1)-treated mice under sham operation and at 6 hours post-I/R.

Data are expressed as mean ± SD, n = 3 per group. Statistical significance was determined using one-way ANOVA followed by Tukey's test for multiple comparisons. *$P < 0.05$, **$P < 0.01$, ***$P < 0.001$.

Table S1. Patient demographic characteristics

Parameters	non-I/R (n=22)	I/R (n=17)	*P* value
Male: Female	18: 4	14 : 3	1.000
Age, years (Mean ± SD)	54.32 ± 14.00	52.41 ± 10.04	0.638
BMI, kg/m^2 (Mean ± SD)	22.37 ± 2.71	23.70 ± 1.91	0.094
Diagnosis			
Hepatocellular carcinoma	17	12	0.569
Cholangiocarcinoma	1	1	
Hepatocellular carcinoma & Cholangiocarcinoma	2	0	
Other liver diseases	2	4	
Operation time, min (Mean ± SD)	177.33 ± 73.62	239.19 ± 64.39	**0.011**
Bleeding volume, ml (Median range)	100 (50, 200)	100 (65, 175)	0.975
Total hepatic inflow occlusion, min (Mean ± SD)	0	46.82 ± 28.69	**0.000**
AST (pre-operation), units/L (Median range)	29.50 (20.00, 35.75)	22.00 (17.50, 26.50)	0.103
ALT (pre-operation), units/L (Median range)	23.50 (14.75, 37.50)	20.00 (14.00, 26.00)	0.600
Total bilirubin (pre-operation), mg/dL (Median range)	13.85 (11.65, 17.40)	13.00 (10.45, 20.40)	0.777
Albumin (pre-operation), g/L (Mean ± SD)	40.52 ± 3.32	38.28 ± 3.79	0.057

Table S2. The targeted sequences of AAVs.

**Name**	**Number**	**Targeted sequence**
Ndufa6 (mouse)	shCON	5′ - GCUGGUCAUUAAGGGAAAGAUTT - 3′
	shNdufa6	5′ - AUCUUUCCCUUAAUGACCAGCTT - 3′

Table S3. The crystal structures of mouse protein

**Name**	**Description**	**Number**
Decr1	2,4-Dienoyl CoA reductase 1	PDB ID: 7UCW
Ahcy	S-adenosylhomocysteine hydrolase	PDB ID: 8COD
Eno1	Enolase 1	NCBI: NP_001366056

Apoc1	Apolipoprotein C1	NCBI: NP_001103479.1
Atp5j	ATP synthase, H^+ transporting, mitochondrial F0 complex, subunit F	NCBI: NP_001289142.1
Cps1	Carbamoyl-phosphate synthetase 1	NCBI: NP_001074278.1
Uqcrfs1	Ubiquinol-cytochrome c reductase, Rieske iron-sulfur polypeptide 1	PDB ID: 7O3E
Cox6c	Cytochrome c oxidase subunit 6C	PDB ID: 8PW5
Dsc1	Desmocollin 1	NCBI: NP_001278733.1
Fabp5	Fatty acid binding protein 5, epidermal	PDB ID: 4AZN
Nccrp1	Non-specific cytotoxic cell receptor protein 1 homolog	NCBI: NP_001074584.2
Fbxl4	F-box and leucine-rich repeat protein 4	NCBI: NP_001411973.1
Gstm1	Glutathione S-transferase, mu 1	NCBI: NP_000552.2
Gapdhs	Glyceraldehyde-3-phosphate dehydrogenase, spermatogenic	PDB ID: 5C7I
Hspa8	Heat shock protein 8	PDB ID: 3CQX
Hbb-b2	Hemoglobin, beta adult minor chain	NCBI: NP_058652.1
Lamb1	Laminin B1	PDB ID: 5MC9
Ndufa6	NADH:ubiquinone oxidoreductase subunit A6	PDB ID: 8C2S
Ybx1	Y box protein 1	NCBI: NP_035862.2
Orc3	Origin recognition complex, subunit 3	NCBI: NP_001153035.1
Prdx2	Peroxiredoxin 2	NCBI: NP_001304314.1
Pik3ca	Phosphatidylinositol-4,5-bisphosphate 3-kinase catalytic subunit alpha	NCBI: NP_032865.2
Ect2	Ect2 oncogene	PDB ID: 2COU
Rbm44	RNA binding motif protein 44	NCBI: NP_001028580.1
Dmkn	Dermokine	NCBI: NP_001159645

Table S4. Primers for qPCR assays.

Gene (Mouse)	qPCR primer sequence
Actb	5' - GATGTATGAAGGCTTTGGTC - 3'<br>5' - TGTGCACTTTTATTGGTCTC - 3'
Acer3	5' - TGTGATTCACTGAGGAACTTTCG - 3'<br>5' - AGAAACTTCACTTTTGGCCTGTA - 3'
Ly6G	5'- GACTTCCTGCAACACAACTACC - 3'

	5' - ACAGCATTACCAGTGATCTCAGT - 3'
Mpo	5' - AGTTGTGCTGAGCTGTATGGA - 3'<br>5' - CGGCTGCTTGAAGTAAAACAGG - 3'
Tnf-α	5' - CCCTCACACTCAGATCATCTTCT - 3'<br>5' - GCTACGACGTGGGCTACAG - 3'
Il-6	5' - TAGTCCTTCCTACCCCAATTTCC - 3'<br>5' - TTGGTCCTTAGCCACTCCTTC - 3'
Ndufa6	5' - TCGGTGAAGCCCATTTTCAGT - 3'<br>5' - CTCGGACTTTATCCCGTCCTT - 3'
**Gene (Human)**	**qPCR primer sequence**
ACTB	5' - CATGTACGTTGCTATCCAGGC - 3'<br>5' - CTCCTTAATGTCACGCACGAT - 3'
ACER3	5' - ACTACTCCGTGACCTGGTACA - 3'<br>5' - GCACCGAACATTGGAGGTATAAT - 3'
NDUFA6	5' - GACGGGATAAAGTCCGAGAAATG - 3'<br>5' - TTCATGGAAGAACCGCATAACA - 3'

Table S5. The list of antibodies.

Antibodies	**Source**	**Identifier**
Anti-ACER3 Rabbit pAb	Sigma-Aldrich	Cat #SAB1303797
Anti-NDUFA6 Rabbit pAb	Novus	Cat #NBP2-93398
Anti-FLAG Rabbit mAb	Abcam	Cat #ab205606
Anti-FACL4 Rabbit mAb	Abcam	Cat #ab155282
Anti-Calreticulin Rabbit mAb	Abcam	Cat #ab92516
Anti-PCNA Rabbit mAb	Cell Signaling Technology	Cat #13110
Anti-VDAC Rabbit mAb	Cell Signaling Technology	Cat #4866
Anti-Cyt C Rabbit mAb	Abcam	Cat #ab133504
Anti-LY6G Rabbit mAb	Abcam	Cat #ab238132
Anti-SMPD3 Mouse pAb	Thermo Fisher Scientific	Cat #PA5-117447
Anti-CERS2 Mouse mAb	Abcam	Cat #ab279372
Anti-β-Actin Rabbit mAb	Cell Signaling Technology	Cat #4970
Anti-GAPDH Rabbit mAb	Cell Signaling Technology	Cat #2118
Anti-rabbit IgG HRP	Cell Signaling Technology	Cat #7074
Anti-mouse IgG HRP	Cell Signaling Technology	Cat #7076
