# Supplementary material for: Ceramide(d18:1/18:1)-NDUFA6 interaction inactivates respiratory complex I to attenuate oxidative-stress-driven pathogenesis in liver ischemia/reperfusion injury
Source: JCI Insight. 2025 Apr 17;10(10):e187083. doi: 10.1172/jci.insight.187083 (PMC12128967; doi:10.1172/jci.insight.187083)

Full unedited gel for Figure 1G:

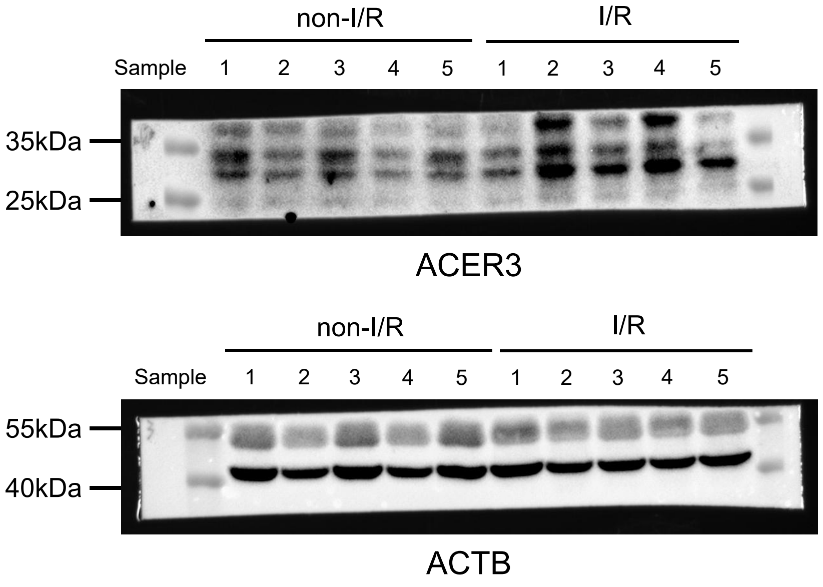

Full unedited gel for Figure 1G:

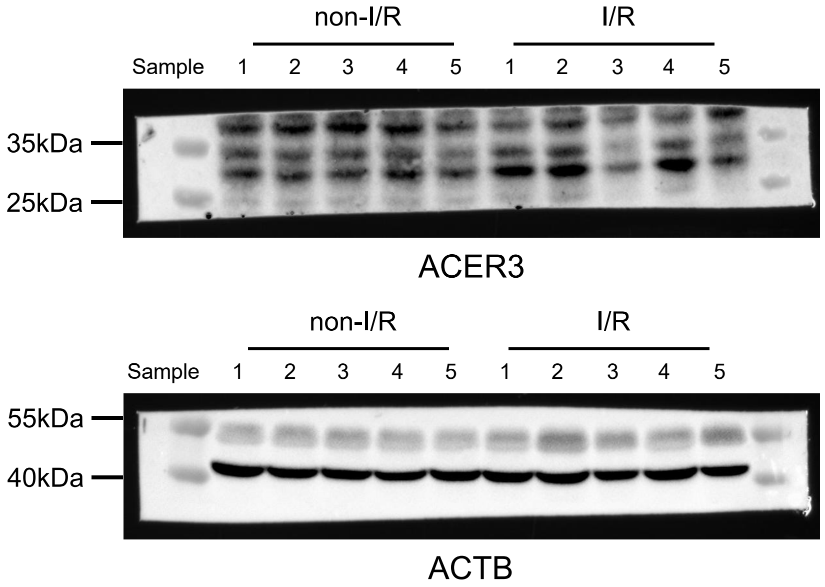

Full unedited gel for Figure 1L:

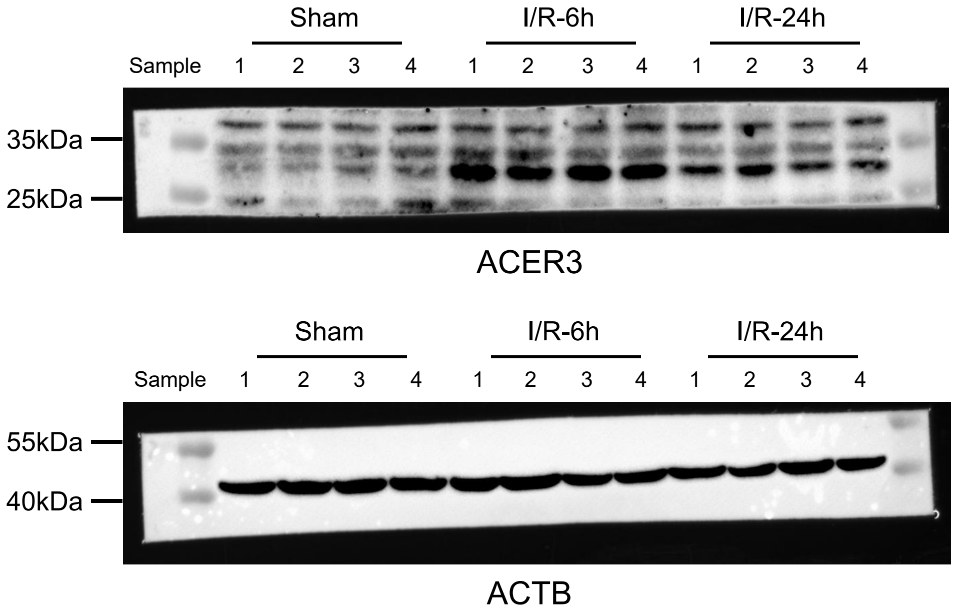

Full unedited gel for Figure 1Q:

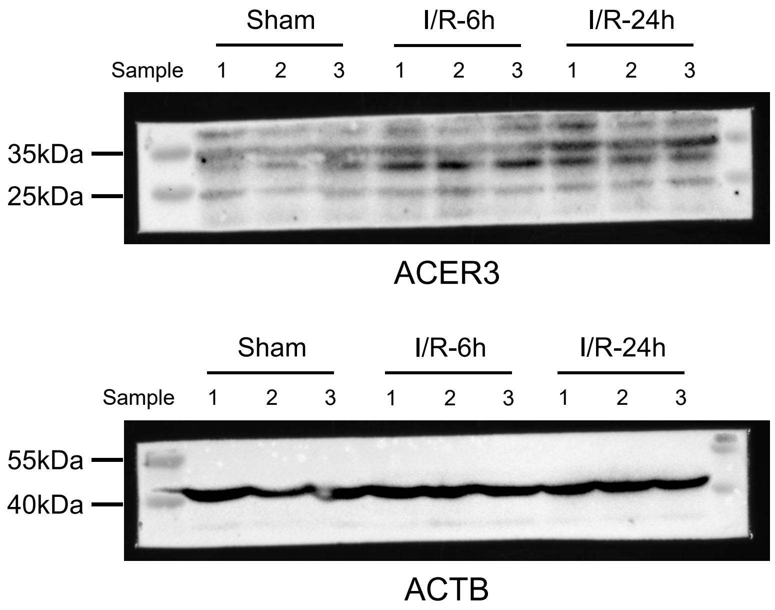

Full unedited gel for Figure 4G:

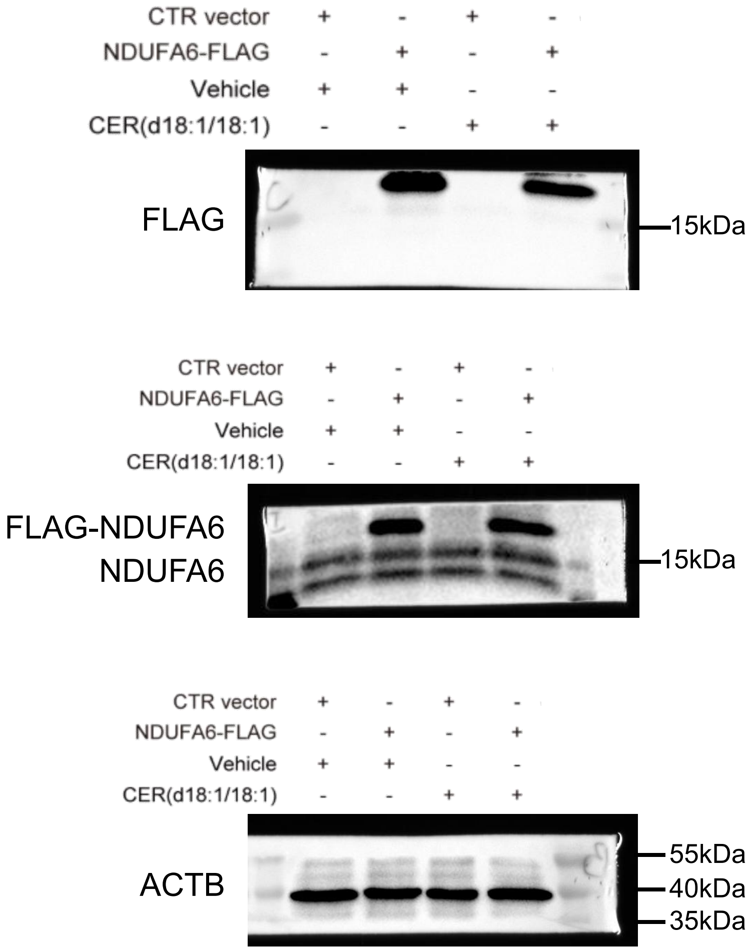

Full unedited gel for Figure 4J:

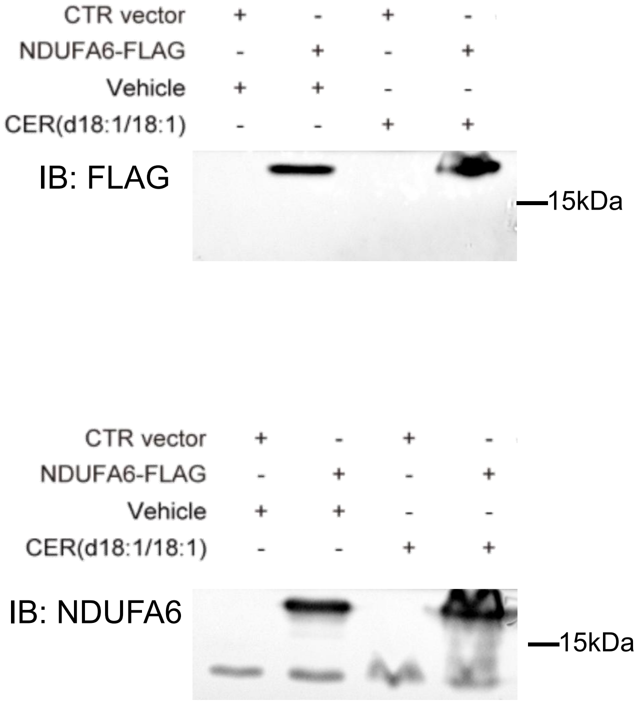

Full unedited gel for Figure 5B:

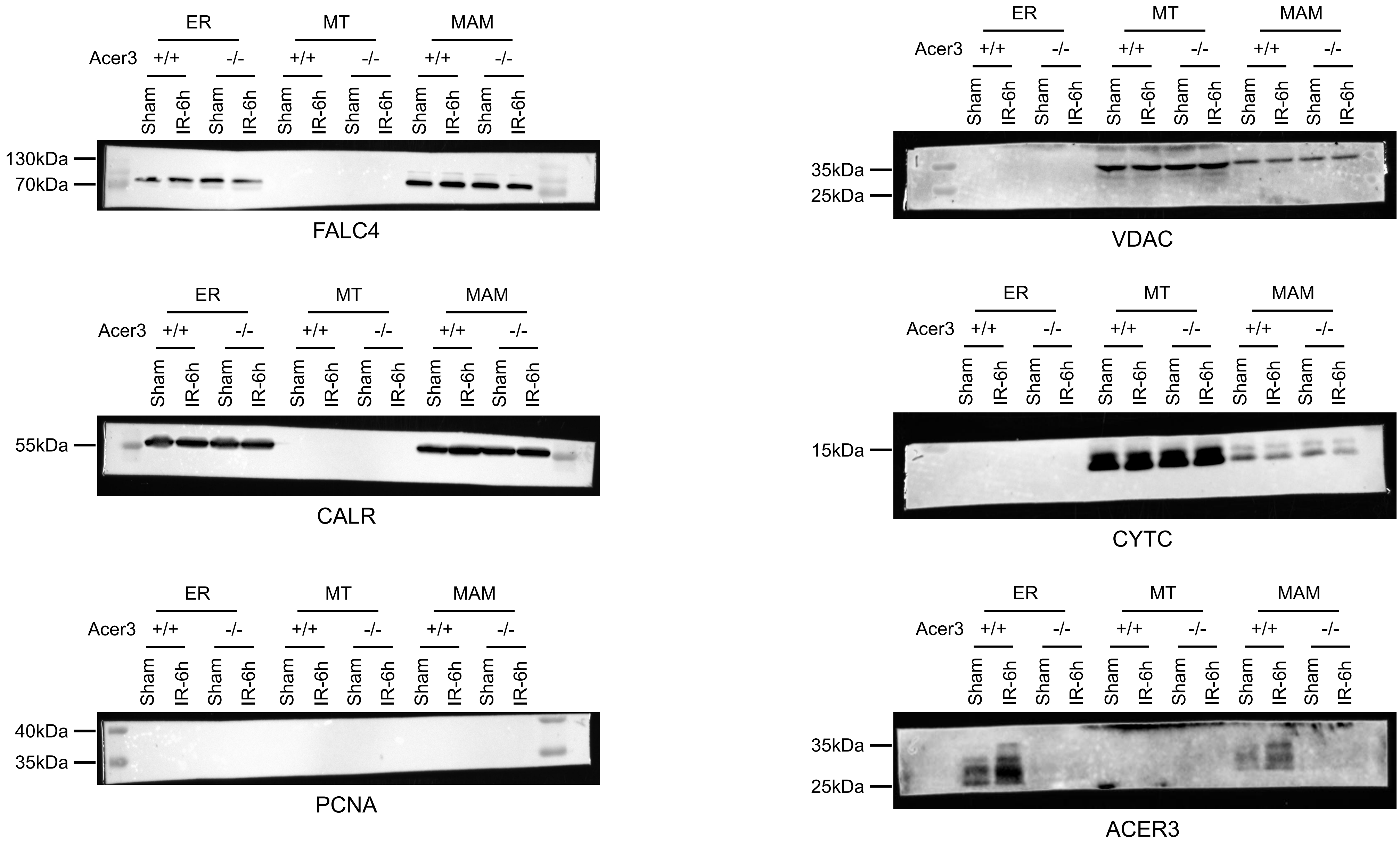

Full unedited gel for Figure 5I:

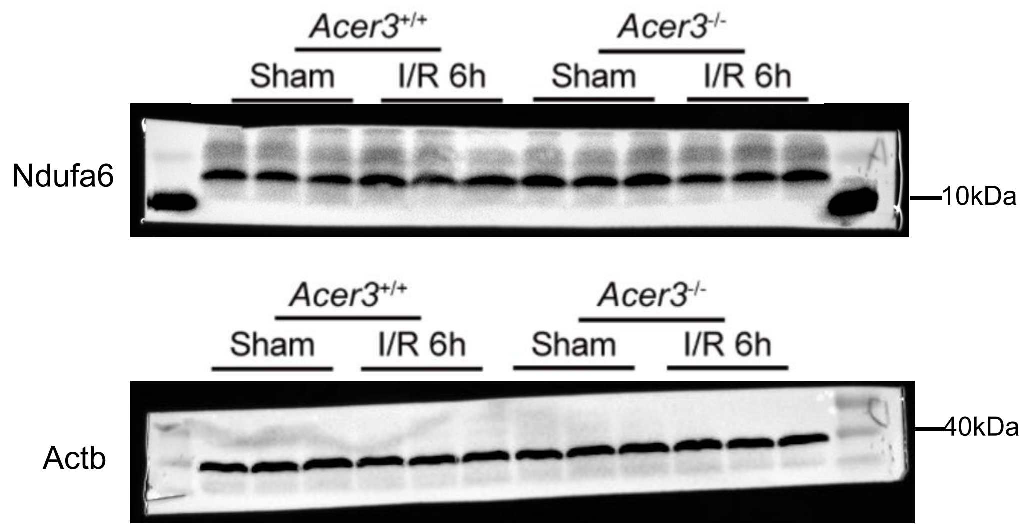

Full unedited gel for Figure 5K:

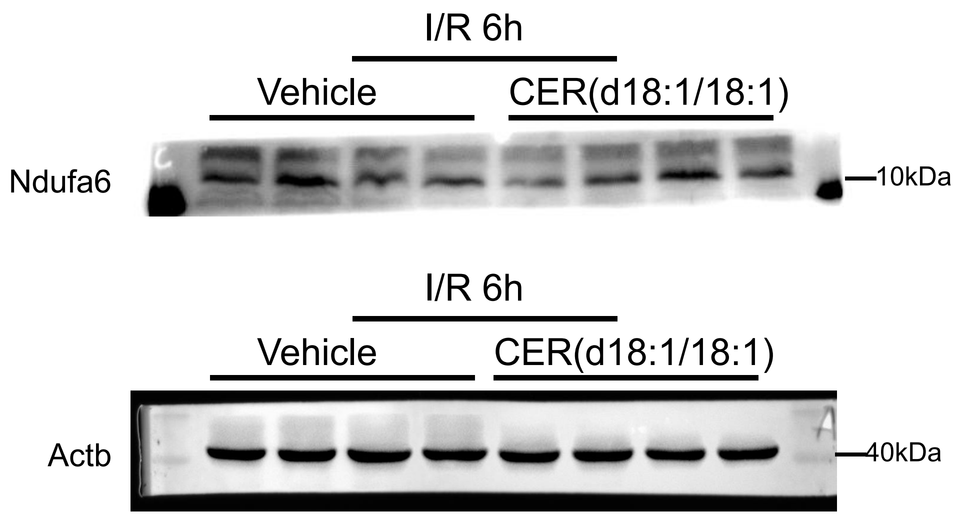

Full unedited gel for Figure 7C:

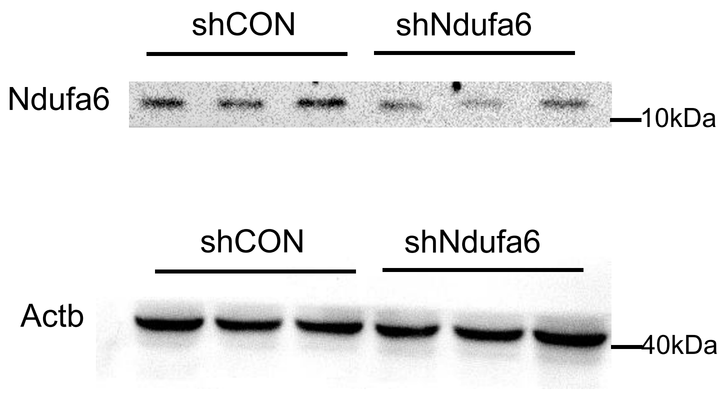

Full unedited gel for Figure 7D:

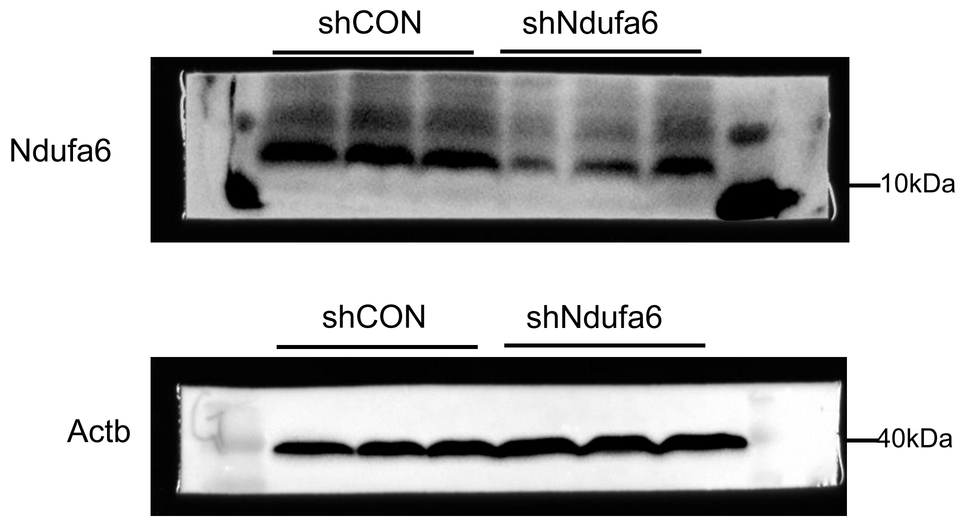

Supplement: Unedited blot and gel images [file jciinsight-10-187083-s127.pdf]
